# Beyond patient education: fall prevention knowledge, health literacy, and implementation gaps in Chinese hospitals—a patient-caregiver study

**DOI:** 10.3389/fpubh.2026.1786050

**Published:** 2026-02-25

**Authors:** Qiannan Zhao, Lingzhu Zhou, Junyan Guo, Yanjun Yang, Shuxiao Hou

**Affiliations:** 1 Department of Nursing Management, Peking University International Hospital, Beijing, China; 2 Department of Nursing Management, School of Nursing, Peking University, Beijing, China

**Keywords:** falls prevention, health literacy, hospitalized older adults, knowledge attitudes behaviors, patient education, patient safety, teach-back method

## Abstract

**Background:**

Falls among hospitalized older adults represent a critical patient safety concern, yet comprehensive assessments of fall prevention knowledge, attitudes, behaviors (KAB), education quality, and health literacy in Chinese hospital settings remain scarce. This study examined these domains and their relationships with fall outcomes.

**Methods:**

This cross-sectional study enrolled patient-caregiver dyads at a tertiary hospital in China between February 2023 and October 2025. Participants completed validated assessments measuring fall prevention knowledge (18-item scale), attitudes (9-item scale), behaviors (14-item scale), education quality metrics (cascade framework with teach-back assessment), health literacy (composite score), and communication barriers. Fall/near-fall events during hospitalization were ascertained through structured interviews. Progressive multivariable logistic regression models examined associations between KAB domains, education quality, health literacy, and fall outcomes.

**Results:**

Among 3,223 participants, 950 (29.5%) reported experiencing at least one fall or near-fall event. Fall prevention knowledge was modest (mean accuracy 58–59%), with critical gaps in recognizing the risks of prior falls (29.6%) and the value of environmental modifications (29.3%). Although attitudes were positive and comparable between groups, caregivers reported significantly higher behavioral engagement than patients (48.80 vs. 45.10; *p* < 0.001); however, adherence to healthcare provider recommendations was notably low across both groups (mean 2.12 ± 1.14/5). The education cascade revealed substantial system attrition: while 69.8% of respondents were informed of fall risks, only 46.6% demonstrated verified comprehension via teach-back. Health literacy was frequently inadequate (41.5% scored ≤2/5), and KAB domains showed negligible intercorrelations (*r* ≤ 0.02). In adjusted multivariable models, KAB scores did not predict fall outcomes. Instead, higher health literacy (aOR = 1.11, 95% CI 1.03–1.19; *p* = 0.006) and greater comfort asking staff questions (aOR = 1.17, 95% CI 1.07–1.27; *p* < 0.001) were independently associated with increased probability of reporting events, despite low overall model discrimination (AUC = 0.577).

**Conclusion:**

Hospital fall prevention requires system-level interventions beyond patient education, including standardized comprehension verification, literacy-sensitive communication, and integration of clinical risk assessment with environmental safety protocols to address multifactorial determinants inadequately captured by KAB-centered frameworks.

## 1 Introduction

Falls among older adults constitute a critical global public health crisis, imposing substantial burden on individuals, healthcare systems, and societies worldwide. Globally, approximately 684,000 fatal falls occur annually, with over 80% occurring in low- and middle-income countries, while an estimated 37.3 million non-fatal falls require medical attention each year (1, 2). Falls represent the second leading cause of unintentional injury death globally and result in more years lived with disability than transport injuries, drowning, burns, and poisoning combined (3, 4). The economic impact is equally staggering, with fall-related healthcare expenditures in high-income countries reaching billions of dollars annually (4). Beyond immediate physical injuries, falls precipitate cascading consequences including reduced functional independence, institutionalization, fear of falling, social isolation, and premature mortality, fundamentally compromising quality of life among affected individuals (5, 6).

Evidence-based fall prevention strategies have been extensively documented in international literature, with multifactorial interventions demonstrating effectiveness in reducing fall incidence among community-dwelling and hospitalized older adults (7, 8). The 2022 World Falls Guidelines provide a comprehensive framework for evidence-based practice, recommending individualized risk assessment, exercise programs, environmental modifications, medication review, and patient education as core components of effective prevention (1). Contemporary risk assessment tools, such as the Fall Risk Assessment Scale developed and validated specifically for rehabilitation hospital patients, provide standardized methods for identifying high-risk individuals and targeting interventions appropriately in acute inpatient settings (9, 10). Systematic reviews have consistently demonstrated that multifactorial interventions can reduce fall rates by 20–30% when properly implemented (11, 12). However, the translation of these evidence-based recommendations into routine clinical practice remains inconsistent across settings and populations, highlighting a persistent research-practice gap (13, 14).

Central to successful fall prevention is the recognition that patients and caregivers play active roles in implementing preventive strategies (15). Knowledge, attitudes, and behaviors (KAB) regarding fall prevention emerge as crucial modifiable factors influencing engagement with evidence-based interventions (16, 17, 89, 90). Studies have revealed that inadequate knowledge about fall risk factors, negative attitudes toward prevention strategies, and suboptimal adoption of protective behaviors significantly impede effective fall prevention (18, 19). Research demonstrates that healthcare providers’ beliefs and attitudes toward fall prevention directly influence their screening practices and patient education delivery, underscoring the importance of comprehensive assessment of KAB across all stakeholders (16, 20, 91). Furthermore, patient education interventions have shown promise in improving fall risk awareness, knowledge levels, and adoption of preventive behaviors among older adults (21).

Health literacy—defined as the capacity to obtain, process, and understand basic health information needed for appropriate health decisions—has emerged as a critical determinant of successful fall prevention engagement (22, 23). Lower health literacy correlates with poor understanding of fall risks, reduced adherence to prevention recommendations, and increased fall rates among older populations (23). Recent scoping reviews highlight the growing recognition of health literacy’s role in fall prevention strategies, yet substantial knowledge gaps persist regarding optimal approaches to deliver education that accommodates varying literacy levels (22, 24). The quality of patient education delivery, including clarity, cultural appropriateness, and utilization of teach-back methods to verify comprehension, significantly influences knowledge retention and behavioral outcomes (25). Despite this recognized importance, systematic assessments of patient education quality and its relationship to fall prevention outcomes remain limited.

China, home to the world’s largest aging population with over 280 million adults aged 60 years and older, faces unprecedented challenges in fall prevention (26). The burden of falls among Chinese older adults has escalated dramatically, with incidence rates increasing substantially from 1990 to 2019, particularly among the oldest-old population (27, 28). Studies report fall prevalence rates ranging from 8.6 to 26.4% among community-dwelling Chinese older adults, varying by geographic region and population characteristics (29, 30). In mainland China, falls constitute the leading cause of injury-related mortality among older adults, yet fall-related disability-adjusted life years (DALYs) have remained relatively stable, suggesting improvements in acute care but persistent prevention gaps (31, 32). The rapid pace of population aging, projected to reach 400 million older adults by 2035, coupled with evolving family structures and increasing empty-nest households, intensifies the urgency for effective fall prevention strategies (33).

Unique contextual factors shape fall prevention challenges in China. Cultural beliefs regarding aging, family caregiving traditions, and stigma associated with acknowledging vulnerability to falls may influence help-seeking behaviors and acceptance of preventive interventions (34, 35). China’s healthcare system, characterized by hospital-centric care delivery and variable integration of preventive services, presents distinct implementation challenges for evidence-based fall prevention programs (36). Furthermore, substantial urban–rural disparities exist in healthcare access, health literacy levels, and fall prevention resource availability, necessitating context-specific approaches (37). Despite increasing recognition of falls as a preventable health issue, systematic evaluations of fall prevention knowledge, attitudes, and behaviors among Chinese hospitalized older adults populations and their caregivers remain scarce (37, 38).

Critical research gaps persist both globally and within the Chinese context regarding the interplay between patient education quality, health literacy, and fall prevention outcomes. While international evidence supports patient education as a fall prevention component, limited research has systematically examined the quality dimensions of education delivery—including clarity, usefulness, confidence-building, and teach-back verification—and their associations with knowledge, attitudes, behaviors, and actual fall outcomes (39, 40). The role of caregivers in fall prevention education has received insufficient attention, despite caregivers’ crucial position in supporting older adults’ daily safety (41, 42). Additionally, communication barriers stemming from limited health literacy have been identified as obstacles to effective fall prevention education, yet few studies have quantitatively assessed these barriers’ relationship to fall risk in hospital settings (40, 43). In China specifically, no studies have comprehensively evaluated the cascade of fall prevention education delivery—from initial risk awareness communication through teach-back assessment—among hospitalized older adults and their family caregivers, nor examined how education quality and health literacy levels relate to fall prevention knowledge, attitudes, behaviors, and fall events.

This single-center cross-sectional study conducted at a tertiary hospital in China aimed to: (1) assess fall prevention knowledge, attitudes, and behaviors among hospitalized older adult patients and their family caregivers; (2) evaluate the quality of fall prevention patient education delivery, health literacy levels, and communication barriers; (3) examine the relationships between education quality, health literacy, KAB domains, and fall or near-fall events; and (4) identify modifiable factors associated with fall risk to inform targeted intervention development for Chinese hospital settings.

## 2 Methodology

### 2.1 Study design and setting

This cross-sectional observational study was conducted at Peking University International Hospital, a tertiary-level academic medical center in Beijing, China, between February 2023 and October 2025. The study employed a dyadic recruitment strategy, enrolling both hospitalized older adult patients and their primary family caregivers as paired respondent units to assess fall prevention knowledge, attitudes, behaviors (KAB), patient education quality, health literacy, and fall-related outcomes (44, 45).

### 2.2 Participant selection and eligibility

#### 2.2.1 Patient inclusion criteria

Eligible patients met all of the following criteria: (1) chronological age ≥65 years at enrollment; (2) hospital admission duration ≥48 h at time of assessment; (3) cognitive capacity to provide informed consent and complete questionnaires reliably, operationally defined as Montreal Cognitive Assessment (MoCA) score ≥18 or absence of documented moderate-to-severe dementia diagnosis in the medical record. The MoCA threshold of ≥18 was selected based on validation studies demonstrating that scores below this cut-point indicate cognitive impairment likely to compromise accurate self-report of knowledge, attitudes, and behaviors (46). For patients without recent MoCA documentation, trained research assistants administered the MoCA during screening. Patients scoring <18 were excluded and their caregivers were not approached for enrollment; (4) functional status permitting verbal communication in Mandarin Chinese; (5) absence of acute medical decompensation requiring intensive care unit-level support; and (6) presence of an identifiable primary family caregiver willing to participate.

#### 2.2.2 Caregiver inclusion criteria

Primary family caregivers were defined as non-professional individuals who: (1) were designated by the patient as their principal source of daily assistance and health-related support; (2) maintained regular (≥3 days per week) contact with the patient; (3) were aged ≥18 years; (4) possessed adequate Mandarin proficiency for questionnaire completion; and (5) provided independent informed consent. Both co-residing and non-co-residing caregivers were eligible provided they fulfilled the primary caregiver role criterion.

#### 2.2.3 Exclusion criteria

Patients were excluded if they exhibited: (1) severe cognitive impairment precluding reliable questionnaire response and informed consent, operationally defined as MoCA score <18 (indicating impairment below the threshold for accurate self-report) or documented moderate-to-severe dementia (Clinical Dementia Rating ≥2 or physician diagnosis of moderate/severe Alzheimer’s disease or vascular dementia in medical records); (2) critical illness with hemodynamic instability; (3) terminal illness with life expectancy <6 months; (4) inability to communicate verbally due to aphasia, severe hearing impairment, or language barriers; or (5) current enrollment in concurrent interventional research protocols. Cognitive screening was conducted by eight trained research assistants who completed standardized MoCA administration training and demonstrated inter-rater reliability (*κ* = 0.89) through double-scoring of 10% of assessments during the pilot phase.

### 2.3 Sample size determination

Sample size calculation employed a two-sample comparison framework based on preliminary institutional data indicating fall prevalence of approximately 18% among hospitalized older adults. Using a significance level (*α*) of 0.05, power (1 − *β*) of 0.80, and anticipated effect size (Cohen’s *h*) of 0.25 for detecting differences in fall prevention knowledge scores between patients and caregivers, the minimum required sample size was calculated as 1,264 patients using G*Power 3.1.9.7 software (47). Accounting for potential incomplete questionnaire data (estimated 10% attrition), the target enrollment was established at 1,400 patient-caregiver dyads. The final recruitment exceeded this target, yielding 3,223 total participants (1,644 patients and 1,579 caregivers), providing >99% power for primary comparative analyses and adequate precision for prevalence estimation; for the inpatient patient sample (*n* = 1,644), a 95% CI half-width is approximately 2.4 percentage points for an event proportion near 0.60.

### 2.4 Recruitment and data collection procedures

#### 2.4.1 Systematic recruitment protocol

Research personnel conducted daily screening of electronic medical records to identify potentially eligible patients across all participating wards. Following preliminary eligibility confirmation, trained research assistants approached patients and their accompanying caregivers during routine hospitalization, typically within 48–72 h post-admission to allow clinical stabilization. After obtaining written informed consent, assessments were conducted via structured face-to-face interviews in private spaces within the hospital ward to ensure confidentiality and minimize environmental distractions.

#### 2.4.2 Data collection timeline and quality control

Data collection occurred during weekday daytime hours (08:00–17:00) to standardize assessment conditions. Each interview session required approximately 30–45 min for patients and 25–35 min for caregivers. Research assistants (*n* = 8) underwent standardized training comprising 16 h of didactic instruction and practical simulation covering questionnaire administration protocols, neutral prompting techniques, and data entry procedures. Inter-rater reliability was assessed through double-coding of 10% of interviews (*κ* = 0.89, indicating excellent agreement). Weekly supervision meetings addressed protocol adherence and resolved interpretative ambiguities.

### 2.5 Measurement instruments and operational definitions

#### 2.5.1 Sociodemographic and clinical characteristics

A structured case report form captured demographic data including age (continuous variable in years), sex (male/female), residential setting (urban/rural classification per National Bureau of Statistics definitions), education level (categorized as: no formal education, primary school, junior middle school, senior high/technical school, college or above), marital status, living arrangements, and health insurance type [Urban Employee Basic Medical Insurance (UEBMI), Urban–Rural Resident Basic Medical Insurance (URRBMI), New Rural Cooperative Medical Scheme (NRCMS)/Integrated rural, commercial insurance, self-pay, or other]. For patients specifically, additional clinical variables included primary admission diagnosis, comorbidity burden (quantified using a modified Charlson Comorbidity Index), hospital length of stay, mobility status, assistive device use, and prescribed medications.

#### 2.5.2 Fall history assessment

Prior fall occurrence was ascertained through direct patient questioning: “In the past 12 months, how many times have you fallen?” Falls were operationally defined following WHO nomenclature as “an event resulting in a person coming to rest inadvertently on the ground or floor or other lower level.” Response categories comprised: (1) zero falls; (2) one fall (single faller); (3) two or more falls (recurrent faller); or (4) uncertain. For patients reporting falls, supplementary data included fall circumstances, injuries sustained, and healthcare utilization resulting from fall events. Time spent alone per 24-h period was categorized as: 0 h, <2 h, 2–4 h, 4–8 h, or >8 h to assess social isolation as a potential fall risk modifier.

#### 2.5.3 Fall prevention knowledge assessment

Fall prevention knowledge was evaluated using an 18-item instrument derived from established fall risk factor literature and validated in Chinese populations (48). Items encompassed intrinsic risk factors (advanced age, medication effects, muscle weakness, vision impairment, balance dysfunction, dizziness, pain interference, urinary urgency, cognitive impairment, fear of falling), extrinsic factors (environmental hazards, inappropriate footwear), and prevention strategies (regular exercise, home modifications, vitamin D supplementation, fall preventability). Each item was scored dichotomously (1 = correct, 0 = incorrect), yielding a composite knowledge score ranging from 0 to 18, with higher scores indicating superior knowledge. Internal consistency reliability in the present sample was acceptable (Cronbach’s *α* = 0.74 for patients, 0.72 for caregivers).

#### 2.5.4 Attitudes toward fall prevention

Attitudes were assessed via a 9-item scale measuring perceptions regarding fall seriousness, prevention importance, personal concern, preventability beliefs, self-efficacy for prevention, healthcare provider responsibility, family involvement necessity, exercise program utility, and environmental modification worthiness. Responses employed a 5-point Likert scale (1 = strongly disagree to 5 = strongly agree), producing a summative attitude score (range: 9–45). Higher scores reflected more favorable fall prevention attitudes. The scale demonstrated satisfactory internal consistency (Cronbach’s *α* = 0.81 patients, 0.79 caregivers) and adequate construct validity through confirmatory factor analysis (CFI = 0.94, RMSEA = 0.063).

#### 2.5.5 Fall prevention behavior engagement

Behavioral engagement was quantified using a 14-item inventory assessing frequency of specific preventive actions: assistive device utilization when required, environmental hazard removal, appropriate footwear selection, regular strength and balance exercise, cautious position changes, keeping frequently-used items accessible, ensuring adequate lighting, installing bathroom safety equipment, medication review with providers, attending scheduled medical appointments, promptly reporting balance disturbances, seeking assistance when needed, adhering to provider recommendations, and participating in fall prevention education (caregiver-specific item). Response options followed a 5-point Likert scale (1 = never to 5 = always). The summative behavior score ranged from 14 to 70 for both patients and caregivers, with elevated scores denoting greater behavioral adoption. Reliability analysis yielded Cronbach’s *α* = 0.85 (patients) and 0.83 (caregivers).

#### 2.5.6 Patient education quality metrics

##### 2.5.6.1 Education cascade assessment

Patient education quality was evaluated through a sequential cascade framework assessing four progressive stages: (1) Fall risk awareness: patients responded whether they had been “told about fall risk” by healthcare providers (yes/no/not sure); (2) Education receipt: whether they “received fall prevention education” (yes/no/not sure); (3) Teach-back request: whether providers asked them to “teach back” or demonstrate understanding of fall prevention information (yes/no); and (4) Teach-back accuracy: self-reported correctness of teach-back response when requested (correct/partly correct/incorrect/not sure). This cascade methodology quantified attrition at each educational stage, illuminating system-level implementation gaps. These items were administered by trained research assistants during structured face-to-face interviews, using standardized question wording developed during pilot testing. Specifically, the teach-back request item asked: “After the healthcare provider explained fall prevention information to you, did they ask you to repeat or explain back the information in your own words?” (response options: Yes/No). For respondents answering “Yes,” the teach-back accuracy item asked: “When you explained the information back, was your response:” (response options: Completely correct/Partly correct/Incorrect/Not sure). Importantly, these assessments captured patient recall and self-perception of teach-back interactions rather than objective, real-time observation of teach-back accuracy by clinical staff. This limitation means that “verified comprehension” in our cascade framework represents patient-reported perceived accuracy rather than clinician-validated understanding. True teach-back effectiveness requires prospective documentation by healthcare providers at the point of care, which was not feasible in this retrospective assessment design. Nevertheless, this cascade methodology quantified patient-perceived attrition at each educational stage, illuminating system-level implementation gaps from the patient and caregiver perspective (49).

##### 2.5.6.2 Education quality dimensions

Among participants receiving education, three quality dimensions were assessed using 5-point Likert scales: (1) Clarity: “How clear was the fall prevention information provided?” (1 = very unclear to 5 = very clear); (2) Perceived usefulness: “How useful did you find this information for preventing falls?” (1 = not at all useful to 5 = extremely useful); and (3) Confidence after education: “How confident do you feel in your ability to prevent falls after receiving this information?” (1 = not at all confident to 5 = extremely confident). These metrics operationalized patient-centered education effectiveness beyond mere information transmission.

#### 2.5.7 Health literacy measurement

##### 2.5.7.1 Single Item Literacy Screener

Functional health literacy was assessed using the validated Single Item Literacy Screener (SILS): “How confident are you filling out medical forms by yourself?” Responses ranged from 1 (not at all confident) to 5 (extremely confident), with scores ≤2 indicating potential limited literacy. This brief screening tool demonstrates strong concurrent validity with comprehensive literacy instruments (AUC = 0.87) and minimizes respondent burden (50).

##### 2.5.7.2 Health literacy composite score

A composite health literacy index was constructed from three validated items: (1) comfort asking healthcare provider questions (1 = not at all comfortable to 5 = extremely comfortable); (2) concern about bothering healthcare providers with questions (1 = not at all concerned to 5 = extremely concerned, reverse-scored); and (3) the SILS item. The composite score (range: 1–5) was calculated as the mean of these three components after appropriate reverse-coding, with higher values indicating superior health literacy. This composite demonstrated adequate internal consistency (Cronbach’s *α* = 0.71) and correlated significantly with education level (*r* = 0.34, *p* < 0.001), supporting construct validity.

#### 2.5.8 Communication barriers assessment

Communication barriers were quantified using a 14-item scale (labeled G1–G14 in study materials) encompassing domains of linguistic complexity, cultural appropriateness, information accessibility, interpersonal communication quality, time constraints, and perceived dismissiveness. Items employed 5-point Likert scaling, generating a summative barriers score (range: 14–70), with elevated scores indicating greater communication obstacles. The instrument exhibited strong reliability (Cronbach’s *α* = 0.88) and correlated inversely with health literacy (*r* = −0.41, *p* < 0.001), supporting construct validity in the expected direction (51).

#### 2.5.9 Fall and near-fall event ascertainment

The primary outcome variable distinguished participants experiencing fall or near-fall events from those without such occurrences during hospitalization (for patients) or as recalled by caregivers regarding the patient’s hospitalization period. Falls were defined per Section 2.5.2 using World Health Organization criteria: “an event resulting in a person coming to rest inadvertently on the ground or floor or other lower level” (52). Near-falls were operationally defined as “situations where you felt you were about to fall but regained balance without actually falling to the ground.” During structured interviews, research assistants first asked: “During this hospitalization, did you (the patient) experience any falls—that is, unintentionally coming to rest on the ground, floor, or other lower level?” (response: Yes/No/Uncertain). Subsequently, they asked: “During this hospitalization, did you (the patient) experience any near-falls—that is, situations where you felt you were about to fall but caught yourself or regained balance without actually falling to the ground?” (response: Yes/No/Uncertain). Respondents answering “Yes” to either question were classified as having experienced an event. For patients reporting events, supplementary data included event circumstances, injuries sustained, and whether medical attention was sought. This composite dichotomous outcome (fall/near-fall occurrence: yes/no) enhanced statistical power by capturing broader fall risk manifestation while maintaining clinical relevance, as near-falls independently predict subsequent injurious falls (53).

### 2.6 Statistical analysis

Data were entered into REDCap (Research Electronic Data Capture) with real-time validation checks and range restrictions. Double data entry was performed for 15% of records to verify accuracy (discrepancy rate: 0.3%). Missing data patterns were evaluated using Little’s MCAR test; variables with <5% missingness were analyzed using complete case analysis, while those exceeding this threshold underwent sensitivity analysis using multiple imputation via chained equations (MICE) with 20 imputations (54). Continuous variables were summarized as means with standard deviations (SD) for normally distributed data or medians with interquartile ranges (IQR) for skewed distributions, following Shapiro–Wilk normality testing. Categorical variables were presented as frequencies and percentages. Between-group comparisons (patients versus caregivers) employed independent samples *t*-tests for normally distributed continuous variables, Mann–Whitney *U* tests for non-normal continuous or ordinal variables, and chi-square tests (or Fisher’s exact tests when expected cell frequencies <5) for categorical variables. Effect sizes were computed using Cohen’s *d* for continuous comparisons and Cramér’s *V* for categorical associations. All hypothesis tests employed two-tailed significance criteria with *α* = 0.05. Relationships among knowledge, attitudes, behaviors (KAB), health literacy, and communication barriers were assessed using Pearson correlation coefficients for continuous normally distributed variables and Spearman rank correlation coefficients for ordinal or non-normally distributed measures. Correlation matrices were constructed to visualize interdomain relationships. The Benjamini–Hochberg false discovery rate (FDR) correction was applied with *q* = 0.05 to control type I error inflation from multiple comparisons.

Initial fall risk factor screening employed univariate binary logistic regression, with fall/near-fall occurrence (yes/no) as the dependent variable. Candidate predictors included demographic characteristics (age, sex, residence, education level), health insurance type, respondent type (patient vs. caregiver), clinical factors (comorbidity count, prior fall history, time alone daily), KAB domain scores, patient education indicators (told about risk, received education, education clarity, education usefulness, confidence after education), and health literacy/communication variables (Single Item Literacy Screener, comfort asking questions, concern about bothering staff, health literacy composite score, communication barriers score). Odds ratios (ORs) with 95% confidence intervals (CIs) were calculated for each predictor. Variables demonstrating associations at *p* < 0.10 in univariate screening were considered for multivariable modeling. A single adjusted multivariable logistic regression model was constructed to evaluate independent associations between patient education, engagement factors, health literacy, and fall/near-fall events while controlling baseline confounders. The adjusted model included: demographic covariates (age, sex, urban residence, education level), insurance type, respondent type, clinical factors (comorbidity count, prior fall history, time alone), KAB scores (knowledge, attitude, behavior), education exposure variables (told about fall risk, received education, education clarity, usefulness), and health literacy/communication variables (health literacy composite, comfort asking questions, concern about bothering staff, communication barriers). Adjusted odds ratios (aORs) with 95% CIs were reported (92).

Model performance was assessed using area under the receiver operating characteristic curve (AUC-ROC), with discrimination classified as: 0.5–0.7 = poor, 0.7–0.8 = acceptable, 0.8–0.9 = excellent, >0.9 = outstanding. Model calibration was evaluated using Hosmer–Lemeshow goodness-of-fit tests. Variance inflation factors (VIF) were computed for all predictors to detect multicollinearity (VIF >5 considered problematic). All regression analyses were conducted in the full respondent sample (*N* = 3,223), with respondent type included as a covariate to account for structural differences between patient and caregiver data. Planned sensitivity analyses addressed: (1) alternative fall outcome definitions (falls only versus falls/near-falls combined) —specifically, restricting analysis to confirmed falls (excluding near-falls) yielded qualitatively similar null associations for KAB predictors, suggesting that inclusion of near-falls did not fundamentally alter interpretations regarding the limited predictive value of education-centric variables; (2) complete case analysis versus multiple imputation for missing data; (3) exclusion of extreme outliers (Cook’s distance >0.5); and (4) bootstrap resampling (1,000 iterations) to verify parameter estimate stability. Subgroup analyses explored effect modification across age categories (65–74, 75–84, ≥85 years), sex, residence type, education level, and health literacy adequacy (SILS score >2 versus ≤2), with interaction terms tested using Bonferroni-corrected significance thresholds. All analyses were performed using Stata 17.0 (StataCorp LLC, College Station, TX) and R version 4.3.1 (R Foundation for Statistical Computing, Vienna, Austria). Graphics were generated using ggplot2 in R and refined using Adobe Illustrator CS6. Statistical significance was set at *p* < 0.05 unless otherwise specified.

## 3 Results

### 3.1 Participant characteristics and fall events

The study enrolled 3,223 participants comprising 1,644 patients (51.0%, mean age 73.1 ± 6.4 years) and 1,579 caregivers (49.0%, mean age 47.8 ± 10.4 years, *p* < 0.001). Sex distribution showed no significant difference between groups (44.2% vs. 45.3% male, *p* = 0.522), nor did urban–rural residence (64.2% vs. 63.6% urban, *p* = 0.756). Educational attainment differed significantly (*p* < 0.001), with caregivers demonstrating higher proportions of college-level education (20.0%) compared to patients (15.4%). Health insurance coverage was universal across both groups, predominantly through Urban Employee Basic Medical Insurance (34.0% patients, 32.8% caregivers) and Urban–Rural Resident Basic Medical Insurance (26.3% patients, 25.0% caregivers). Patient-specific clinical characteristics revealed a mean comorbidity burden of 3.47 ± 1.24 conditions. Among patients, 35.9% reported previous falls, 58.7% denied fall history, and 5.4% were uncertain (caregivers were not assessed for personal fall history). Daily time spent alone varied considerably across the full cohort, with 19.6% never alone, 26.4% alone for less than 2 h, 22.9% for 2–4 h, 20.9% for 4–8 h, and 10.0% alone for more than 8 h daily. Overall, 950 respondents (29.5%) reported at least one fall or near-fall event, whereas 2,273 (70.5%) reported no event.

### 3.2 Knowledge, attitudes, and behaviors assessment

Composite knowledge scores (range 0–18) demonstrated no significant difference between patients (10.56 ± 2.01) and caregivers (10.46 ± 2.03, *p* = 0.143), representing approximately 58–59% overall accuracy. Item-level analysis revealed substantial heterogeneity in knowledge domains, with participants demonstrating highest proficiency in recognizing muscle weakness (66.1%), vision problems (64.4%), and dizziness (64.8%) as fall risk factors. However, critical knowledge gaps were identified: only 29.6% correctly recognized that previous falls increase future fall risk, 29.3% understood the effectiveness of home modifications, and 28.3% recognized vitamin D deficiency as a risk factor. Between-group comparisons identified only two significant differences: patients demonstrated superior recognition of environmental hazards (56.3% vs. 51.5%, *p* = 0.022) and vision problems (66.4% vs. 62.3%, *p* = 0.019) as fall risk factors. Scale reliability was acceptable with Cronbach’s *α* of 0.74 for patients and 0.72 for caregivers.

Attitudes toward fall prevention, measured on a 9–45 point scale, were remarkably similar between groups: patients scored 30.63 ± 3.58 and caregivers scored 30.63 ± 3.63 (*p* = 0.981), representing approximately 68% of maximum possible endorsement. All nine attitude items demonstrated no between-group differences (all *p* > 0.20). Participants expressed strong agreement that healthcare providers should discuss fall risks (mean scores 3.51–3.53 on 5-point scales), that falls are serious problems (3.48–3.54), and that fall prevention is important (3.45–3.50). However, attitudes toward environmental modifications received the lowest endorsement (2.77–2.79), suggesting potential barriers to implementing home safety measures. Scale reliability was good with Cronbach’s *α* of 0.81 for patients and 0.79 for caregivers.

Fall prevention behaviors revealed the study’s most significant finding. Caregivers reported substantially higher behavioral engagement (48.80 ± 4.31) compared to patients (45.10 ± 4.08, *p* < 0.001), representing an 8.2% relative difference and a medium-to-large effect size (Cohen’s *d* = 0.88). This translated to approximately 70% versus 64% of maximum possible behavioral adoption among caregivers and patients, respectively (48.80/70 = 69.7% vs. 45.10/70 = 64.4%). Individual preventive behaviors demonstrated uniformly high adoption rates (mean scores 3.52–3.64 on 5-point scales) across most domains including appropriate footwear use, environmental hazard removal, adequate lighting maintenance, assistive device utilization, and cautious position changes. However, a critical implementation gap was identified: adherence to healthcare provider recommendations scored markedly lower (mean 2.11–2.13), equivalent to only 42% adoption despite universally positive attitudes toward provider guidance. Scale reliability was good with Cronbach’s *α* of 0.85 for patients and 0.83 for caregivers.

### 3.3 Patient education quality and health literacy

Sequential assessment of the patient education cascade revealed substantial attrition across four stages based on patient and caregiver retrospective self-report of educational interactions. Among total participants, 69.8% (*n* = 2,251) reported being told about fall risks by healthcare providers, with no significant difference between patients (69.4%) and caregivers (70.3%, *p* = 0.464). Among those told about risk (*n* = 2,251), 95.7% (*n* = 2,155) subsequently received formal structured education, representing 4.3% attrition from initial awareness communication. Overall, 66.9% of the total sample received formal education. Critically, only 51.8% reported that teach-back was requested, whereas 34.1% reported it was not requested; the remaining respondents were uncertain or had missing data for this item, and therefore the proportion without comprehension verification should not be inferred solely as 100 to 51.8%. Among the total sample, teach-back responses (including those not asked) were distributed as: 46.6% fully correct, 27.6% partially correct, 14.2% incorrect, and 11.6% unsure. Among only those asked to teach-back (*n* = 1,671), 89.9% reported fully correct responses. Calculating from initial awareness communication to self-reported fully correct teach-back status at the whole-sample level, 46.6% reported fully correct responses; among those who were asked to teach back (*n* = 1,671), 89.9% reported fully correct responses. However, these teach-back accuracy data reflect patient self-assessment of their performance rather than objective clinician verification at the time of education delivery, potentially leading to overestimation of comprehension due to recall bias and social desirability.

Education quality dimensions, assessed among those who received formal education, demonstrated moderate ratings with no between-group differences. Clarity of information averaged 3.65 ± 1.14 for patients and 3.68 ± 1.15 for caregivers (*p* = 0.449), perceived usefulness averaged 3.71 ± 1.16 and 3.77 ± 1.13, respectively, (*p* = 0.175), and confidence after education averaged 3.59 ± 1.17 and 3.55 ± 1.16 (*p* = 0.336). These scores represented approximately 73–74% of maximum possible ratings. Health literacy assessment using the Single Item Literacy Screener (1–5 scale, where ≤2 indicates inadequate literacy) revealed mean scores of 2.83 ± 1.38 for patients and 2.86 ± 1.36 for caregivers (*p* = 0.571), positioning both groups slightly above but near the inadequacy threshold (reference value = 2.0). Distribution analysis showed 42.3% of patients and 40.8% of caregivers scored in the inadequate range (≤2), with an overall prevalence of limited literacy at 41.5% across the full sample. The three-component health literacy composite (incorporating SILS, comfort asking questions, and concern about bothering providers) scored identically at 3.00 ± 0.74 for patients and 3.00 ± 0.76 for caregivers (*p* = 0.890). Communication barriers, measured on a 14–70 scale, averaged 48.04 ± 4.42 for patients and 47.99 ± 4.43 for caregivers (*p* = 0.760), indicating moderate perceived obstacles to effective healthcare communication.

**Figure 1:**
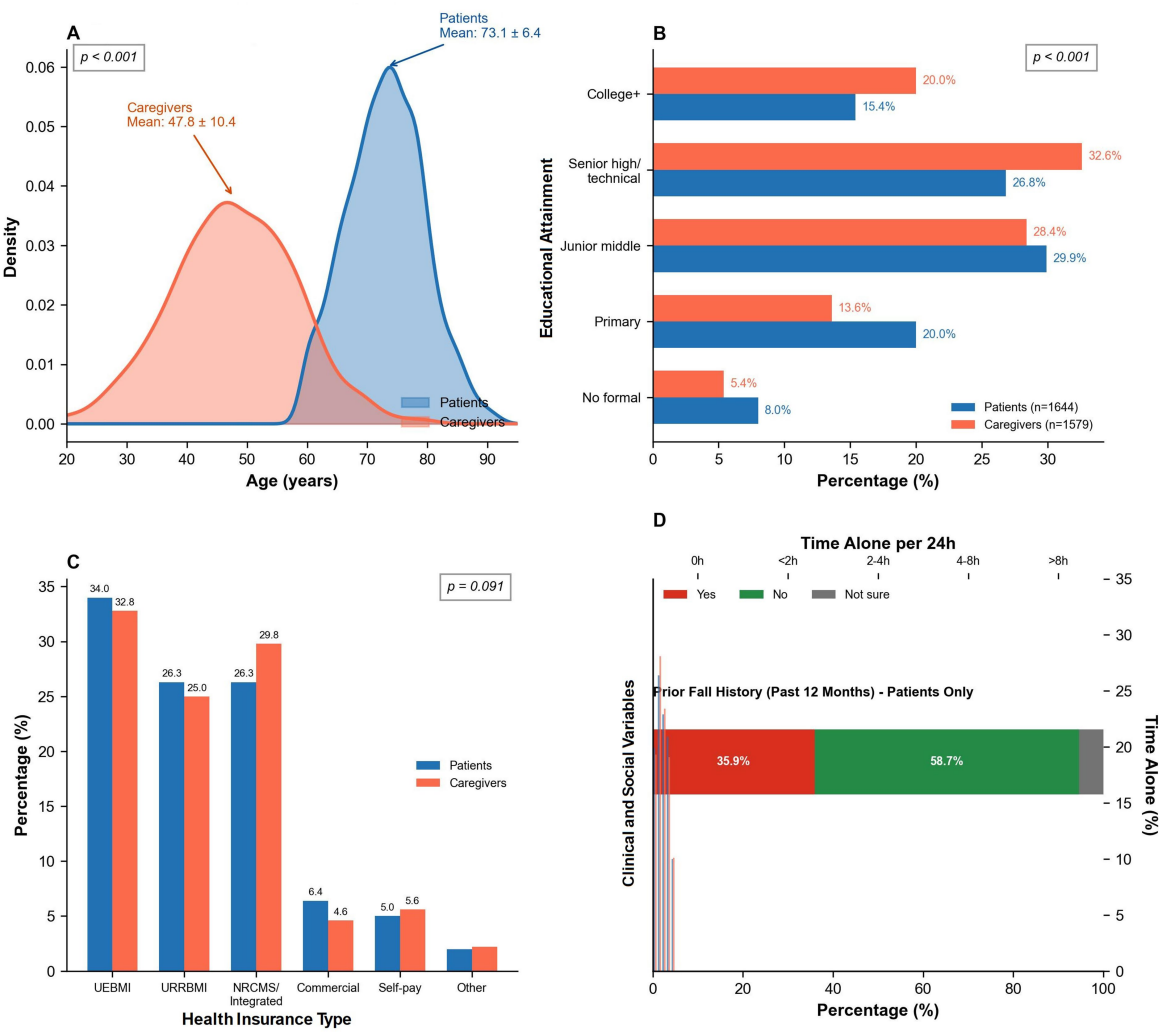
Study population demographics and clinical characteristics (*N* = 3,223). **(A)** Age distribution by respondent type. **(B)** Education level distribution. **(C)** Health insurance coverage. **(D)** Prior fall history & time alone.

**Figure 2:**
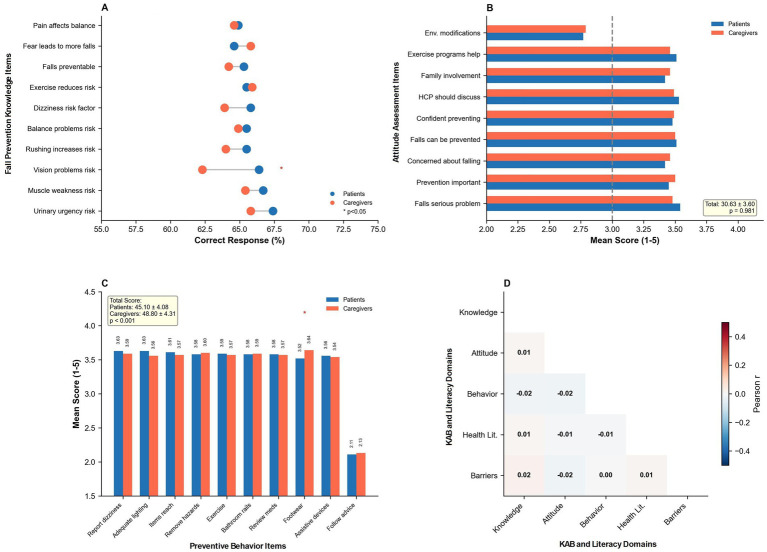
Fall prevention knowledge, attitudes, and behaviors. **(A)** Knowledge item performance. **(B)** Attitudes toward fall prevention. **(C)** Fall prevention behavior engagement. **(D)** KAB domain correlations.

**Figure 3:**
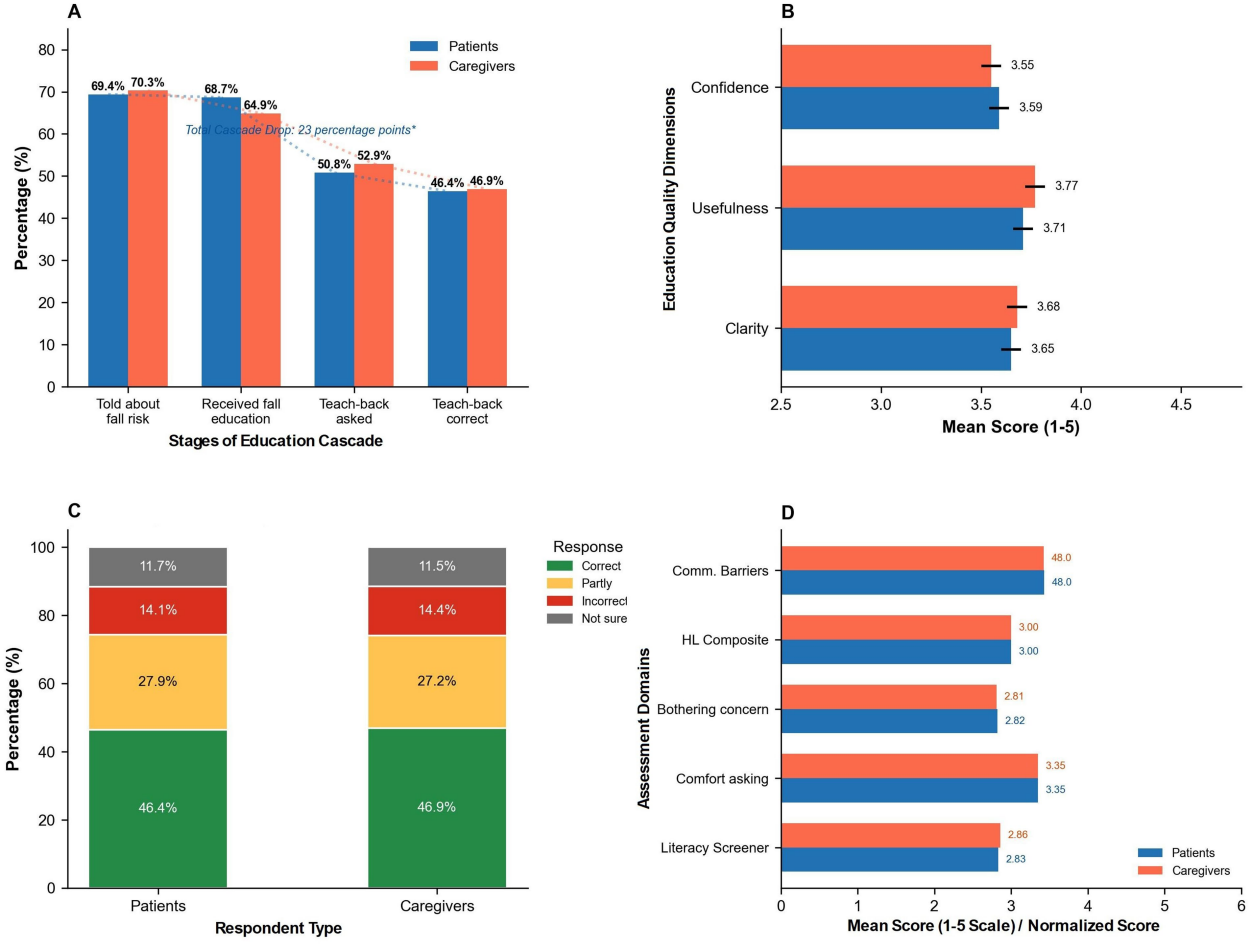
Patient education quality and health literacy assessment. **(A)** Fall prevention education cascade. **(B)** Education quality assessment. **(C)** Teach-back response accuracy (full sample, *N* = 3,223). **(D)** Health literacy and communication barriers.

**Figure 4:**
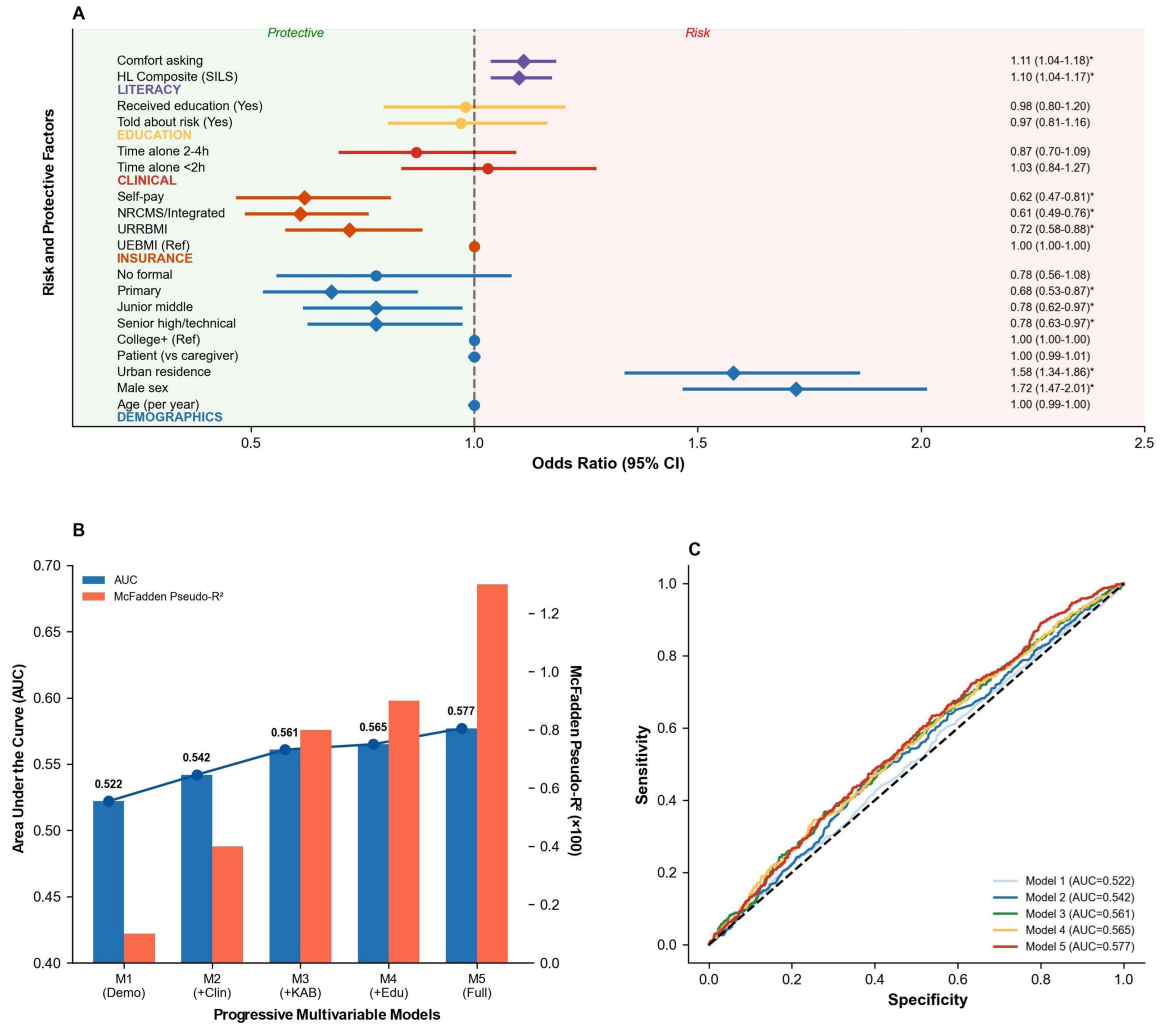
Risk factor analysis and predictive model performance. **(A)** Univariate analysis of fall/near-fall risk factors (Tables 6, 7 univariate). **(B)** Progressive model performance (sequential addition of covariates). **(C)** ROC curves for progressive models. Models utilize full dyadic sample (*N* = 3,223).

Correlation analysis revealed a theory-challenging finding: all knowledge-attitude-behavior-health literacy domains demonstrated weak-to-negligible intercorrelations (all |*r*| ≤ 0.02, all *p* > 0.05). Specifically, knowledge-attitudes (*r* = 0.01), knowledge-behaviors (*r* = −0.02), attitudes-behaviors (*r* = −0.02), health literacy-knowledge (*r* = 0.01), health literacy-attitudes (*r* = −0.01), and health literacy-behaviors (*r* = −0.01) showed essentially zero association. After Benjamini–Hochberg false discovery rate correction (*q* = 0.05), no correlations achieved statistical significance. These findings challenge the conventional assumption that knowledge, attitudes, behaviors, and health literacy function as sequentially related or mutually reinforcing components within a unified theoretical framework for fall prevention. Instead, they suggest that these constructs operate independently in the acute inpatient context, with important implications for intervention design and theoretical model development in hospital-based fall prevention research (see Figures 1–4; Tables 1–5).

**Table 1:** Baseline demographic and clinical characteristics by respondent type.

Characteristic	Total (*N* = 3,223)	Patients (*n* = 1,644)	Caregivers (*n* = 1,579)	*p*-value
Total participants	3,223 (100.0)	1,644 (51.0)	1,579 (49.0)	
Age, mean (SD)	60.7 (15.3)	73.1 (6.4)	47.8 (10.4)	<0.001
Sex, *n* (%)				
Male	1,442 (44.7)	726 (44.2)	716 (45.3)	
Female	1,781 (55.3)	918 (55.8)	863 (54.7)	0.522
Residence, *n* (%)				
Urban	2,059 (63.9)	1,055 (64.2)	1,004 (63.6)	
Rural	1,164 (36.1)	589 (35.8)	575 (36.4)	0.756
Education level, *n* (%)				
No formal	216 (6.7)	131 (8.0)	85 (5.4)	
Primary	544 (16.9)	329 (20.0)	215 (13.6)	
Junior middle	939 (29.1)	491 (29.9)	448 (28.4)	
Senior high/Technical	955 (29.6)	440 (26.8)	515 (32.6)	
College+	569 (17.7)	253 (15.4)	316 (20.0)	<0.001
Health insurance, *n* (%)				
UEBMI	1,077 (33.4)	559 (34.0)	518 (32.8)	
URRBMI	827 (25.7)	432 (26.3)	395 (25.0)	
NRCMS/Integrated rural	903 (28.0)	433 (26.3)	470 (29.8)	
Commercial	178 (5.5)	105 (6.4)	73 (4.6)	
Self-pay	171 (5.3)	82 (5.0)	89 (5.6)	
Other	67 (2.1)	33 (2.0)	34 (2.2)	0.091
Comorbidity count, mean (SD)	3.47 (1.24)	3.47 (1.24)	—	—
Prior fall in past 12 months, *n* (%)				
Yes	591 (18.3)	591 (35.9)	0 (0.0)	
No	965 (29.9)	965 (58.7)	0 (0.0)	
Not sure	88 (2.7)	88 (5.4)	0 (0.0)	
Unknown	1,579 (49.0)	0 (0.0)	1,579 (100.0)	<0.001
Time alone per 24 h, *n* (%)				
0 h	631 (19.6)	327 (19.9)	304 (19.3)	
<2 h	878 (27.2)	434 (26.4)	444 (28.1)	
2–4 h	745 (23.1)	376 (22.9)	369 (23.4)	
4–8 h	645 (20.0)	343 (20.9)	302 (19.1)	
>8 h	324 (10.1)	164 (10.0)	160 (10.1)	0.669

*p*-values from chi-square tests for categorical variables and independent *t*-tests for continuous variables. SD, standard deviation; UEBMI, Urban Employee Basic Medical Insurance; NRCMS, New Rural Cooperative Medical Scheme; URRBMI, Urban–Rural Resident Basic Medical Insurance.

**Table 2:** Fall risk knowledge assessment by respondent type (correct responses).

Knowledge item	Total (*N* = 3,223) *n* (%)	Patients (*n* = 1,644) *n* (%)	Caregivers (*n* = 1,579) *n* (%)	*p*-value
Age is a risk factor for falls	2,085 (64.7)	1,050 (63.9)	1,035 (65.5)	0.289
Medications can increase fall risk	2,102 (65.2)	1,048 (63.7)	1,054 (66.8)	0.197
Environmental hazards contribute to falls	1,739 (54.0)	926 (56.3)	813 (51.5)	0.022
Muscle weakness increases fall risk	2,129 (66.1)	1,096 (66.7)	1,033 (65.4)	0.746
Vision problems increase fall risk	2,075 (64.4)	1,092 (66.4)	983 (62.3)	0.019
Balance problems increase fall risk	2,101 (65.2)	1,077 (65.5)	1,024 (64.9)	0.911
Rushing increases fall risk	2,087 (64.8)	1,076 (65.5)	1,011 (64.0)	0.057
Dizziness is a fall risk factor	2,090 (64.8)	1,081 (65.8)	1,009 (63.9)	0.208
Previous falls increase future fall risk	955 (29.6)	486 (29.6)	469 (29.7)	0.993
Pain affects balance	2,087 (64.8)	1,067 (64.9)	1,020 (64.6)	0.864
Poor footwear increases fall risk	2,073 (64.3)	1,069 (65.0)	1,004 (63.6)	0.457
Urinary urgency increases fall risk	2,147 (66.6)	1,108 (67.4)	1,039 (65.8)	0.521
Cognitive impairment increases fall risk	2,046 (63.5)	1,033 (62.8)	1,013 (64.2)	0.599
Fear of falling can lead to more falls	2,101 (65.2)	1,062 (64.6)	1,039 (65.8)	0.743
Regular exercise reduces fall risk	2,116 (65.7)	1,076 (65.5)	1,040 (65.9)	0.887
Home modifications can prevent falls	944 (29.3)	468 (28.5)	476 (30.1)	0.054
Vitamin D deficiency increases fall risk	913 (28.3)	475 (28.9)	438 (27.7)	0.24
Falls are preventable	2,087 (64.8)	1,074 (65.3)	1,013 (64.2)	0.054
Total knowledge score (0–18), mean (SD)	10.51 (2.02)	10.56 (2.01)	10.46 (2.03)	0.143

Values represent number and percentage of correct responses. *p*-values from chi-square tests for individual items and independent *t*-test for total knowledge score. Higher scores indicate better knowledge (range 0–18).

**Table 3:** Attitudes toward fall prevention by respondent type.

Attitude item	Total (*N* = 3,223)	Patients (*n* = 1,644)	Caregivers (*n* = 1,579)	*p*-value
Falls are a serious health problem	3.51 (1.19)	3.54 (1.16)	3.48 (1.22)	0.15
Fall prevention is important	3.47 (1.20)	3.45 (1.19)	3.50 (1.20)	0.26
I am concerned about falling	3.44 (1.21)	3.42 (1.20)	3.46 (1.23)	0.32
Falls can be prevented	3.51 (1.20)	3.51 (1.20)	3.50 (1.21)	0.848
I feel confident in preventing falls	3.49 (1.20)	3.48 (1.19)	3.49 (1.20)	0.83
Healthcare providers should discuss fall risks	3.51 (1.23)	3.53 (1.20)	3.49 (1.26)	0.45
Family involvement is important in fall prevention	3.44 (1.19)	3.42 (1.19)	3.46 (1.19)	0.402
Exercise programs can help prevent falls	3.49 (1.20)	3.51 (1.18)	3.46 (1.23)	0.216
Environmental modifications are worthwhile	2.78 (1.30)	2.77 (1.30)	2.79 (1.30)	0.627
Total attitude score (9–45), mean (SD)	30.63 (3.60)	30.63 (3.58)	30.63 (3.63)	0.981

Values are mean (SD) on a 5-point Likert scale (1 = strongly disagree, 5 = strongly agree). *p*-values from independent *t*-tests. Higher scores indicate more positive attitudes toward fall prevention (range 9–45).

**Table 4:** Fall prevention behaviors and engagement by respondent type.

Behavior item	Total (*N* = 3,223)	Patients (*n* = 1,644)	Caregivers (*n* = 1,579)	*p*-value
Use assistive devices when needed	3.55 (1.16)	3.56 (1.15)	3.54 (1.16)	0.52
Remove environmental hazards	3.59 (1.15)	3.58 (1.16)	3.60 (1.14)	0.63
Wear appropriate footwear	3.58 (1.15)	3.52 (1.16)	3.64 (1.14)	0.002
Exercise regularly for strength/balance	3.58 (1.15)	3.59 (1.15)	3.57 (1.16)	0.601
Take time when getting up or changing position	3.57 (1.17)	3.55 (1.17)	3.59 (1.16)	0.363
Keep frequently used items within reach	3.59 (1.16)	3.61 (1.14)	3.57 (1.18)	0.425
Ensure adequate lighting	3.60 (1.15)	3.63 (1.13)	3.56 (1.16)	0.085
Use bathroom rails and grab bars	3.58 (1.15)	3.58 (1.17)	3.59 (1.13)	0.89
Review medications with healthcare provider	3.57 (1.15)	3.58 (1.13)	3.57 (1.17)	0.943
Attend scheduled medical appointments	3.56 (1.16)	3.57 (1.16)	3.55 (1.16)	0.632
Report dizziness or balance problems	3.61 (1.14)	3.63 (1.13)	3.59 (1.15)	0.402
Ask for help when needed	3.58 (1.16)	3.59 (1.15)	3.58 (1.16)	0.748
Follow healthcare provider recommendations	2.12 (1.14)	2.11 (1.13)	2.13 (1.15)	0.572
Total behavior score (14–70), mean (SD)	46.92 (4.59)	45.10 (4.08)	48.80 (4.31)	<0.001

Values are mean (SD) on a 5-point Likert scale (1 = never, 5 = always). *p*-values from independent *t*-tests. Higher scores indicate more frequent engagement in fall prevention behaviors (range 14–70).

**Table 5:** Patient education quality, health literacy, and communication barriers by respondent type.

Metric	Total (*N* = 3,223)	Patients (*n* = 1,644)	Caregivers (*n* = 1,579)	*p*-value
Told about fall risk, *n* (%)				
Yes	2,251 (69.8)	1,141 (69.4)	1,110 (70.3)	
No	596 (18.5)	300 (18.2)	296 (18.7)	
Not sure	376 (11.7)	203 (12.3)	173 (11.0)	0.464
Received fall prevention education, *n* (%)				
Yes	2,155 (66.9)	1,130 (68.7)	1,025 (64.9)	
No	718 (22.3)	340 (20.7)	378 (23.9)	
Not sure	350 (10.9)	174 (10.6)	176 (11.1)	0.054
Clarity of education (1–5)	3.67 (1.15)	3.65 (1.14)	3.68 (1.15)	0.449
Perceived usefulness (1–5)	3.74 (1.15)	3.71 (1.16)	3.77 (1.13)	0.175
Confidence after education (1–5)	3.57 (1.16)	3.59 (1.17)	3.55 (1.16)	0.336
Teach-back asked, *n* (%)				
Yes	1,671 (51.8)	835 (50.8)	836 (52.9)	
No	1,100 (34.1)	555 (33.8)	545 (34.5)	0.057
Teach-back response category (whole sample), *n* (%)				
Yes	1,502 (46.6)	762 (46.4)	740 (46.9)	
Partly	888 (27.6)	458 (27.9)	430 (27.2)	
No	459 (14.2)	232 (14.1)	227 (14.4)	
Not sure	374 (11.6)	192 (11.7)	182 (11.5)	0.975
Single Item Literacy Screener (1–5)	2.85 (1.37)	2.83 (1.38)	2.86 (1.36)	0.571
Comfort asking questions (1–5)	3.35 (1.26)	3.35 (1.26)	3.35 (1.26)	0.995
Bothering concern (1–5)	2.81 (1.27)	2.82 (1.26)	2.81 (1.28)	0.718
Health literacy composite score	3.00 (0.74)	3.00 (0.73)	3.00 (0.76)	0.89
Communication barriers score (14–70)	48.02 (4.42)	48.04 (4.42)	47.99 (4.43)	0.76

Values are *n* (%) for categorical variables and mean (SD) for continuous variables. *p*-values from chi-square tests for categorical variables and independent *t*-tests for continuous variables. Higher scores indicate better education quality, higher health literacy, and more communication barriers (G1–G14 items).

### 3.4 Fall risk factor analysis

In univariable logistic regression, distinct sociodemographic profiles emerged as correlates of fall/near-fall reporting. Male sex (OR = 1.72, 95% CI 1.47–2.01; *p* < 0.001) and urban residence (OR = 1.58, 95% CI 1.34–1.86; *p* < 0.001) were associated with significantly higher odds of reported events. Conversely, participants with lower educational attainment (primary to senior high school) and those covered by non-employee insurance schemes (URRBMI, NRCMS, Self-pay) demonstrated lower odds of reporting events compared to college-educated and UEBMI-insured reference groups (all *p* < 0.05) (Table 6). Respondent type (caregiver vs. patient) and time spent alone were not statistically associated with event status.

**Table 6:** Univariable associations between baseline characteristics and fall/near-fall events.

Characteristic	Fall/Near-fall (*n* = 950)	No fall/Near-fall (*n* = 2,273)	OR (95% CI)	*p*-value
Age, years	60.4 (15.0)	60.8 (15.4)	1.00 (0.99–1.00)	0.527
Sex: Female (ref)	434 (45.7%)	1,345 (59.2%)	1.00 (ref)	—
Sex: Male	516 (54.3%)	928 (40.8%)	1.72 (1.47–2.01)	<0.001
Residence: Rural (ref)	275 (28.9%)	889 (39.1%)	1.00 (ref)	—
Residence: Urban	675 (71.1%)	1,384 (60.9%)	1.58 (1.34–1.86)	<0.001
Education: College+ (ref)	254 (26.7%)	491 (21.6%)	1.00 (ref)	—
Education: Senior high/Technical	237 (24.9%)	590 (26.0%)	0.78 (0.63–0.97)	0.026
Education: Junior middle	244 (25.7%)	602 (26.5%)	0.78 (0.62–0.97)	0.029
Education: Primary	151 (15.9%)	432 (19.0%)	0.68 (0.53–0.87)	0.003
Education: No formal	64 (6.7%)	158 (7.0%)	0.78 (0.56–1.08)	0.131
Insurance: UEBMI (ref)	299 (31.5%)	540 (23.8%)	1.00 (ref)	—
Insurance: URRBMI	233 (24.5%)	588 (25.9%)	0.72 (0.58–0.88)	0.002
Insurance: NRCMS/Integrated rural	220 (23.2%)	648 (28.5%)	0.61 (0.49–0.76)	<0.001
Insurance: Commercial	79 (8.3%)	144 (6.3%)	0.99 (0.73–1.34)	0.955
Insurance: Self-pay	93 (9.8%)	272 (12.0%)	0.62 (0.47–0.81)	0.001
Insurance: Other	26 (2.7%)	81 (3.6%)	0.58 (0.37–0.91)	0.018
Respondent type: Patient (ref)	486 (51.2%)	1,158 (50.9%)	1.00 (ref)	—
Respondent type: Caregiver	464 (48.8%)	1,115 (49.1%)	0.99 (0.85–1.15)	0.867
Time alone in past 24 h: 0 h (ref)	220 (23.2%)	511 (22.5%)	1.00 (ref)	—
Time alone in past 24 h: <2 h	258 (27.2%)	582 (25.6%)	1.03 (0.84–1.27)	0.76
Time alone in past 24 h: 2–4 h	195 (20.5%)	522 (23.0%)	0.87 (0.70–1.09)	0.22
Time alone in past 24 h: 4–8 h	155 (16.3%)	356 (15.7%)	1.01 (0.79–1.28)	0.95
Time alone in past 24 h: >8 h	122 (12.8%)	302 (13.3%)	0.94 (0.72–1.22)	0.627

Baseline sociodemographic, insurance, respondent-type, and recent supervision characteristics associated with self-reported fall or near-fall events among study respondents (*N* = 3,223). Values are presented as mean (SD) for continuous variables and *n* (%) for categorical variables. Odds ratios (ORs) with 95% confidence intervals (CIs) were estimated using binary logistic regression with the outcome defined as any fall or near-fall event (yes vs. no); reference categories are indicated as “ref.” *p*-values correspond to Wald tests for individual regression coefficients, and two-sided *p*-values are reported, with *p* < 0.05 indicating statistical significance. These estimates quantify the univariable associations between baseline characteristics and event status, thereby informing covariate selection for multivariable models and interpretation of risk gradients.

In adjusted analyses controlling for baseline covariates (Table 7), specific engagement and literacy factors showed independent associations with outcomes. Higher health literacy scores were independently associated with increased odds of reporting events (aOR = 1.11, 95% CI 1.03–1.19; *p* = 0.006). Furthermore, greater comfort in asking staff questions significantly predicted higher reporting (aOR = 1.17, 95% CI 1.07–1.27; *p* < 0.001). However, formal education exposure variables—including being told about risk, receiving education, and the perceived clarity or usefulness of that education—did not retain statistical significance after adjustment. Despite identifying these independent predictors, the overall predictive models demonstrated weak discrimination. Model performance metrics improved marginally with the addition of variables (Model 1 AUC = 0.522 to Model 5 AUC = 0.577), and the full model explained only a small proportion of variance (McFadden pseudo-*R*^2^ = 0.013). This indicates that while sociodemographic factors, health literacy, and communication comfort are statistically significant correlates, they alone do not sufficiently account for the multifactorial nature of fall risk in this inpatient setting.

**Table 7:** Patient-centered education and engagement factors associated with fall/near-fall events.

Characteristic	Fall/near-fall (*n* = 950)	No fall/near-fall (*n* = 2,273)	Unadjusted OR (95% CI)	*p*	Adjusted OR (95% CI)	*p* ^adj^
Told about fall risk: No (ref)	208 (21.9%)	486 (21.4%)	1.00 (ref)	—	1.00 (ref)	—
Told about fall risk: Yes	658 (69.3%)	1,591 (70.0%)	0.97 (0.81–1.16)	0.74	1.04 (0.85–1.28)	0.683
Told about fall risk: Not sure	84 (8.8%)	196 (8.6%)	1.00 (0.75–1.34)	0.987	1.02 (0.74–1.41)	0.909
Received fall-prevention education: No (ref)	175 (18.4%)	414 (18.2%)	1.00 (ref)	—	1.00 (ref)	—
Received fall-prevention education: Yes	718 (75.6%)	1,735 (76.3%)	0.98 (0.80–1.20)	0.856	0.93 (0.74–1.17)	0.541
Received fall-prevention education: Not sure	57 (6.0%)	124 (5.5%)	1.09 (0.78–1.53)	0.621	1.18 (0.81–1.72)	0.388
Education clarity score (1–5)	3.60 (0.92)	3.61 (0.91)	0.99 (0.92–1.06)	0.742	0.99 (0.91–1.07)	0.739
Education usefulness score (1–5)	3.68 (0.94)	3.69 (0.94)	0.99 (0.93–1.06)	0.819	0.95 (0.87–1.03)	0.202
Confidence in preventing falls (1–5)	3.53 (0.95)	3.54 (0.95)	0.99 (0.93–1.06)	0.796	1.06 (0.97–1.15)	0.188
Health literacy (SILS; 1–5)	2.39 (0.95)	2.30 (0.95)	1.10 (1.04–1.17)	0.002	1.11 (1.03–1.19)	0.006
Comfort asking staff questions (1–5)	3.59 (0.95)	3.50 (0.95)	1.11 (1.04–1.18)	0.002	1.17 (1.07–1.27)	<0.001
Concern about bothering staff (1–5)	2.56 (0.99)	2.54 (0.99)	1.02 (0.97–1.08)	0.434	0.99 (0.92–1.06)	0.704

Associations of patient-centered fall-prevention education exposure, perceived quality of education, engagement-related perceptions, and health literacy with self-reported fall or near-fall events among study respondents (*N* = 3,223). Values are presented as mean (SD) for Likert-scale items and *n* (%) for categorical education-exposure items; higher Likert scores denote greater endorsement of the construct as coded in the questionnaire. Unadjusted and adjusted ORs with 95% CIs were derived from binary logistic regression models with fall/near-fall status (yes vs. no) as the dependent variable; adjusted models simultaneously included the education/engagement variables shown and controlled for baseline covariates specified in the Methods (e.g., age, sex, residence, education level, insurance type, respondent type, and recent time alone), with reference categories denoted as “ref.” *p*-values are two-sided Wald tests, and statistical significance was defined a priori as *p* < 0.05. These findings delineate whether education delivery and engagement factors exhibit independent associations with reported events after accounting for baseline differences.

Adjusted ORs were derived from a multivariable logistic regression model controlling for age, sex, urban residence, education level, insurance type, respondent type, comorbidity count, prior fall history, time alone, knowledge score, attitude score, behavior score, fall risk awareness, education receipt, education clarity, education usefulness, health literacy composite, and communication barriers score. *N* = 3,223 for all analyses.

## 4 Discussion

Falls and near-falls among hospitalized older adults remain a clinically consequential and system-relevant safety problem, particularly in aging societies where hospital-based care is increasingly tasked with both acute management and prevention. In this large single-center cross-sectional study of 3,223 respondents (1,644 hospitalized patients aged ≥65 years and 1,579 family caregivers), several findings were notable. First, despite pronounced age differences between respondent types, patients and caregivers displayed broadly comparable fall-prevention knowledge and attitudes, whereas caregivers reported substantially higher self-reported preventive behaviors. Second, the fall-prevention education process exhibited marked attrition at the stage of comprehension verification, with only approximately half of respondents reporting teach-back, and fewer than half achieving fully correct teach-back responses. Third, health literacy was frequently limited, communication barriers were moderate, and—counter to prevailing assumptions—knowledge, attitudes, behaviors, health literacy, and barriers were essentially uncorrelated.

A central observation was that overall knowledge about fall risk factors and prevention strategies was modest and highly heterogeneous across specific items, with substantial gaps in core determinants that are typically emphasized in guideline-based risk communication. Although aggregate knowledge scores were similar between patients and caregivers (approximately 58–59% accuracy), recognition of prior falls as a strong predictor of future falls and understanding of home modification benefits were each below one-third of respondents. These deficits are clinically consequential, because prior falls represent a cornerstone of risk stratification and trigger multifactorial assessment in international and national guidance, including the World Guidelines for Falls Prevention and Management, which highlight prior falls and environmental hazards as actionable determinants of subsequent risk (1). Notably, the pattern of uneven knowledge aligns with scoping and systematic reviews showing that older adults frequently recognize generic intrinsic risk factors (e.g., weakness, dizziness) but under-recognize the prognostic value of previous falls and the effectiveness of specific preventive strategies (e.g., home hazard modification, vitamin D where indicated) (18, 21). In contrast, the similarity between patients and caregivers in overall knowledge challenges a frequent assumption that caregivers inherently possess superior fall-prevention literacy; rather, caregivers may be exposed to the same episodic, non-tailored health communication as patients during hospitalization, yielding parallel knowledge profiles.

Attitudinal profiles in the present study were uniformly moderate-to-positive, with little between-group variation and high endorsement that falls are serious, that prevention matters, and that clinicians should discuss fall risk. This configuration is consistent with broader literature showing that positive general attitudes toward fall prevention can coexist with incomplete understanding of risk determinants and limited uptake of specific recommendations, particularly when educational content is generic or when perceived feasibility is low (16, 18). The comparatively low endorsement of environmental modification as worthwhile is also concordant with cultural and practical barriers reported among older Chinese adults and families, including beliefs about aging, concerns about household disruption, and constrained resources for home adaptations (34, 37). In settings where hospital discharge planning and community support are variable, environmental modification may be viewed as outside the immediate locus of control, thereby weakening attitudinal commitment even when falls are perceived as serious (37).

The most pronounced between-group difference was behavioral engagement, with caregivers reporting higher preventive behaviors than patients (total behavior score 48.80 ± 4.31 vs. 45.10 ± 4.08; *p* < 0.001; Cohen’s *d* = 0.88). In principle, this finding is plausible because caregivers often function as the operational agents of prevention—managing environments, supervising mobility, and facilitating care navigation—whereas hospitalized older adults may have restricted mobility, acute illness, or functional limitations that reduce autonomous engagement. Qualitative studies from hospital contexts similarly emphasize that caregivers often assume responsibility for safety behaviors, although they frequently report insufficient tailored education and limited opportunity to clarify uncertainties with health professionals (15, 55). However, the present results also reveal a critical implementation discordance: adherence to provider recommendations was markedly low (mean ≈2.1/5 = 42% adoption) in both groups despite positive attitudes toward clinician involvement and despite caregivers reporting high engagement in nearly all other preventive behaviors (mean scores 3.5–3.6/5 = 70–72% adoption). This juxtaposition suggests that “agreement” with prevention principles does not translate to adoption of clinician-directed actions, potentially because recommendations are perceived as impractical, insufficiently explained, inconsistently reinforced, or competing with acute care priorities (56, 57). This “performance-practice gap”—wherein respondents perceive themselves as actively engaged in fall prevention yet simultaneously report poor adherence to the specific recommendations provided by healthcare professionals—has several plausible explanations. First, the measured behaviors (e.g., wearing appropriate footwear, ensuring adequate lighting, using assistive devices) may represent autonomous safety actions that caregivers and patients implement based on common sense or prior experience, whereas “following provider recommendations” refers specifically to clinician-directed advice that may be perceived as impractical, poorly explained, or misaligned with the patient’s functional status or home environment. Second, social desirability bias may inflate self-reported engagement in generic preventive behaviors while respondents feel more comfortable honestly disclosing non-adherence to provider advice. Third, provider recommendations may be delivered as generic, non-tailored instructions (e.g., “be careful getting up,” “remove hazards at home”) that lack actionable specificity, reducing perceived feasibility and motivation for adoption. Finally, the low adherence may reflect insufficient shared decision-making during education delivery—recommendations that are not co-developed with patients and families may be viewed as externally imposed mandates rather than collaboratively designed strategies, undermining ownership and follow-through. These mechanisms converge on the conclusion that high self-reported engagement in fall prevention behaviors does not guarantee alignment with evidence-based professional guidance, underscoring the need for patient-centered education approaches that prioritize actionable, context-specific recommendations delivered through collaborative dialogue rather than unidirectional information transmission. Such an implementation gap aligns with evidence from process evaluations and qualitative work indicating that fall-prevention messages are often delivered briefly, with limited shared decision-making, and without structured reinforcement or verification of comprehension (14, 55).

A major contribution of this study is the operationalization of fall-prevention education as a cascade, enabling identification of the principal attrition points. Approximately 70% of respondents reported being told about fall risk, two-thirds reported receiving education, but only about half reported that teach-back was used. Moreover, at the whole-sample level, 46.6% reported fully correct teach-back responses; among those who were asked to teach back (*n* = 1,671), 89.9% reported fully correct responses. This pattern is consistent with prior observations that educational delivery is commonly documented as “provided” without systematic assessment of understanding, and that teach-back—despite its established role in improving comprehension across health contexts—is not routinely implemented due to time constraints, workflow limitations, and variable staff training (25, 49, 58). Recent qualitative work underscores that even when education is delivered, patients and caregivers may perceive it as generic and insufficiently personalized to their functional status or environment, which may reduce salience and retention (59, 60). Importantly, the education quality ratings (clarity, usefulness, confidence) were moderate and similar across respondent types, indicating that perceived “quality” alone may not capture whether critical content was delivered, tailored, or retained (61).

The findings also intersect with emerging literature emphasizing that hospital fall prevention is not solely dependent on patient education but on a multi-layered safety system that includes staff practices, environmental design, and organizational culture. Education is a necessary component of multifactorial interventions in many frameworks, but its effect may be conditional on concurrent structural supports, such as safe staffing, standardized risk assessment tools, mobility protocols, medication review processes, and environmental hazard controls (1, 7, 39). Therefore, the observed cascade attrition likely reflects system-level implementation constraints rather than individual-level unwillingness. These results suggest that institutional efforts should prioritize routinized comprehension verification and actionable discharge-oriented counseling, rather than assuming that risk notification and information provision are sufficient.

Health literacy is increasingly recognized as a determinant of preventive engagement in older adults, and prior work has linked limited health literacy to poorer understanding of fall risk and reduced adherence to preventive recommendations (22, 23). In this study, however, mean health literacy screening scores hovered near the inadequacy threshold, and approximately two-fifths of both groups fell into the limited-literacy range. Furthermore, perceived communication barriers were moderate. Notably, despite these gradients, correlations among knowledge, attitudes, behaviors, health literacy, and barriers were essentially null (|*r*| ≤ 0.02). This finding represents a theory-challenging observation that contradicts the foundational assumptions underlying many fall prevention education interventions. Conventional KAB models implicitly assume that knowledge gains lead to attitude changes, which in turn motivate behavioral adoption—a sequential pathway that should generate positive intercorrelations among domains (62, 63). The complete absence of such associations in our inpatient sample suggests that KAB constructs, while validated in community-based settings where individuals have autonomy and time to implement changes, do not function as an integrated framework in acute hospital environments. This has critical implications: educational interventions targeting knowledge alone are unlikely to modify behaviors or attitudes in hospitalized older adults, and effective inpatient fall prevention requires fundamentally different theoretical models that account for institutional constraints, acute clinical factors, and the limited agency patients have over their immediate environment during hospitalization.

Several scientific explanations may account for this apparent disconnect. First, measurement non-equivalence across respondent types and across constructs may attenuate observed correlations. Knowledge items were scored dichotomously, whereas attitudes and behaviors were Likert-based, and these different scaling properties can reduce linear associations, especially when distributions are narrow (e.g., attitudes clustering around similar values) (64, 65). Second, inpatient environments constrain behavioral expression; even when knowledge or attitudes are present, institutional routines and patient functional status may dominate behavior, obscuring relationships that might be more evident in community settings. Third, caregivers’ reported behaviors may reflect intention or perceived responsibility rather than directly observed actions, introducing social desirability and recall bias that can weaken associations with knowledge and attitudes (66, 67). Fourth, the content delivered through education may not map directly onto the specific knowledge items assessed, limiting the extent to which education quality relates to knowledge or behavior outcomes. Finally, contemporary fall risk is multifactorial and may be more strongly influenced by acute illness severity, delirium, medication changes, mobility limitations, and ward environment than by KAB constructs, particularly over short time horizons (1, 68).

Perhaps the most clinically provocative result was the positive association between health literacy, communication comfort, and reported fall/near-fall outcomes. Unlike standard clinical models where fall risks are often inversely related to health literacy, the adjusted analysis (Table 7) showed that individuals with higher health literacy (aOR = 1.11, 95% CI 1.03–1.19; *p* = 0.006) and greater comfort asking questions (aOR = 1.17, 95% CI 1.07–1.27; *p* < 0.001) were more likely to report events. This finding almost certainly reflects reporting and detection bias rather than any causal relationship between literacy and fall risk. Specifically, patients with higher health literacy and communication comfort are more capable of recognizing ambiguous events (e.g., brief loss of balance, stumbling, grabbing for support) as reportable “near-falls,” whereas individuals with limited literacy may dismiss identical events as non-incidents or lack the vocabulary to articulate them when questioned. This interpretation is further supported by the inclusion of near-falls in the outcome definition—events that are inherently subjective and require cognitive recognition and linguistic articulation to be captured in self-report assessments. Importantly, this mechanism implies that the observed association represents differential event detection and reporting, not differential event occurrence, and causal interpretations would be inappropriate. For readers unfamiliar with inpatient fall dynamics, it is critical to recognize that higher literacy facilitates accurate reporting of events that actually occurred, rather than increasing actual fall risk—a distinction with important implications for interpreting fall surveillance data and designing literacy-appropriate assessment protocols. This may reflect a “reporting bias” where more literate and engaged individuals are better at recognizing and articulating near-miss events, whereas those with lower literacy may under-report. Alternatively, highly engaged patients may be more mobile and independent, paradoxically increasing their exposure to risk scenarios. Moreover, discrimination remained poor even in the full model (AUC = 0.577), and pseudo-*R*^2^ values indicated that the models explained only a small fraction of outcome variance. This low predictive utility highlights a theoretical mismatch: while KAB constructs are distal determinants suitable for predicting long-term community trends, they lack explanatory power for acute inpatient falls driven by proximal, dynamic triggers. These findings contrast with some community-based studies in which lower health literacy, weaker prevention knowledge, and poorer adoption of safety behaviors correlate with higher fall risk (21, 69). However, they align with the broader evidence base that, in hospital settings, falls are heavily influenced by clinical acuity, medications (particularly psychoactive drugs), gait instability, delirium, toileting urgency, staffing and supervision, environmental layout, and timing factors—domains not fully captured by the present KAB-centered framework (70, 71). Critically, the consistently low model discrimination (maximum AUC = 0.577) reflects not merely statistical insufficiency but a fundamental conceptual mismatch: the distal determinants measured in this study (knowledge, attitudes, behaviors, education exposure, perceived quality) are unlikely to exert short-term causal effects on fall events that are predominantly driven by acute clinical instability, rapidly changing medication regimens, delirium, severe mobility impairment, and immediate environmental hazards. This theoretical limitation underscores that KAB frameworks, while relevant for community-based prevention where individuals have autonomy over long-term behavioral adoption, have limited explanatory power in acute inpatient rehabilitation settings where proximal clinical and environmental factors dominate risk pathways over hours to days rather than weeks to months.

The counterintuitive direction of the univariate health literacy signal warrants careful interpretation. The most parsimonious explanation is detection and reporting bias: individuals with higher literacy possess superior capacity to recognize ambiguous balance disturbances as “near-falls,” articulate these events using appropriate medical terminology, and feel comfortable disclosing them during structured interviews—whereas individuals with limited literacy may experience identical physiological events but dismiss them as inconsequential or lack the language to describe them accurately. This mechanism is particularly relevant given the subjective nature of near-fall definitions and the reliance on self-report outcome ascertainment in this study. A plausible explanation is reverse causality: patients who experience a fall or near-fall may have more contact with clinicians, receive more explanations, and consequently report higher confidence in interacting with healthcare systems, artificially inflating health literacy scores (72, 73). Critically, these interpretations converge on the conclusion that the observed positive association does not reflect a causal effect of literacy on fall risk, but rather differential event detection, reporting, or post-event healthcare engagement. Readers should interpret these findings as evidence that literacy influences how fall events are recognized and communicated, not whether they occur. Alternatively, higher literacy may correlate with greater mobility and independence, which could increase exposure to fall opportunities in a hospital environment. Another explanation is detection or reporting bias: individuals with higher literacy may better recognize and report near-fall events, whereas those with lower literacy may under-report. Finally, residual confounding by unmeasured clinical factors (e.g., gait impairment, sedative exposure, delirium) could generate a spurious positive association in unadjusted analysis (74, 75). These interpretations are consistent with the well-recognized limitations of cross-sectional designs for causal inference and underscore the need for prospective designs with standardized outcome ascertainment (76).

While age, comorbidity count, and time spent alone showed no association, male sex and urban residence emerged as strong predictors of fall events in this cohort (*p* < 0.001). This contrasts with global literature often citing female preponderance in falls but may reflect risk-taking behaviors or mobility patterns among male inpatients in this setting (72, 77). Moreover, prior fall history was assessed among patients, but the models’ restriction to patients for higher-order models may limit power to detect modest effects when predictor distributions are constrained (78, 79). Additionally, the narrow age range among patients (mean 73.1 ± 6.4 years) reduces variance, diminishing the detectable age effect compared with studies spanning wider adult age ranges.

Although the present study found limited evidence that KAB domains and education quality metrics directly predict fall/near-fall events, the data nonetheless identify actionable targets for improving patient-centered prevention in Chinese hospital settings. First, the education cascade highlights a clear, modifiable system failure: comprehension verification via teach-back is inconsistently implemented (used with only ~52% of respondents), and among the total sample, only 46.6% achieved fully correct teach-back responses. Hospitals should implement the following system-level interventions to address this gap: (1) Embed mandatory teach-back verification into electronic medical record (EMR) workflows by creating a structured documentation field that requires nurses to record whether teach-back was attempted, the patient’s response accuracy (correct/partial/incorrect), and any re-education provided before the fall risk assessment can be electronically signed and closed. This EMR-based forcing function ensures teach-back becomes a non-optional component of fall prevention education rather than a discretionary practice dependent on individual clinician initiative. (2) Implement structured nurse training protocols that include didactic instruction on teach-back methodology, simulated practice sessions with standardized patients, competency assessment through direct observation of at least three teach-back interactions, and ongoing quality monitoring through monthly audits of EMR documentation with feedback to nursing units. Training should emphasize that teach-back is not “testing” the patient but rather verifying that the clinician’s explanation was clear and understandable. (3) Develop literacy-stratified education materials tailored to the 41.5% of patients with limited health literacy identified in this study, including visual aids depicting fall risk factors and prevention strategies (e.g., pictorial guides showing proper use of assistive devices, bathroom safety equipment, appropriate footwear), simplified language scripts for verbal education (avoiding medical jargon and using action-oriented phrasing), and availability of interpreter support for patients with language barriers beyond Mandarin proficiency. These materials should be pilot-tested with low-literacy patient advisory groups to ensure comprehensibility. Embedding teach-back into routine workflows—particularly for high-risk patients—could improve knowledge retention, align expectations, and facilitate safer transitions to home. Embedding teach-back into routine workflows—particularly for high-risk patients—could improve knowledge retention, align expectations, and facilitate safer transitions to home. Teach-back implementation is feasible within existing nursing and allied health roles and has demonstrated effectiveness in improving patient understanding and reducing readmissions in other clinical contexts (80, 81). Importantly, successful implementation likely requires structured training and organizational support, consistent with staff perspectives emphasizing that brief but frequent education, supported by institutional culture, is essential (82).

Second, the pattern of knowledge deficits suggests that education content should be more strategically targeted. Emphasis on the prognostic importance of prior falls, the benefits and practicalities of environmental modification, and the role of vitamin D and strength/balance interventions (where clinically appropriate) may address the largest knowledge gaps. Notably, these topics align with international guidelines and high-quality evidence syntheses supporting multifactorial interventions and exercise-based approaches for fall prevention (7, 83). Third, the discordance between positive attitudes and low adherence to provider recommendations implies that education should prioritize actionable, context-specific behaviors and shared decision-making, rather than general messaging. In practice, this may involve bedside demonstrations, mobility planning, toileting strategies, medication counseling, and family-inclusive safety planning tailored to the patient’s functional status and home environment (84, 85).

Fourth, the high prevalence of limited health literacy underscores the need for literacy-sensitive communication. Health literacy-informed approaches—including simplified language, pictorial aids, repetition, and opportunities for questions—may improve comprehension and engagement, even if health literacy was not an independent predictor of events in this cross-sectional design. Furthermore, addressing perceived communication barriers may require clinician training in patient-centered communication and time allocation for education, consistent with literature documenting limited dialogue between professionals, older adults, and caregivers about fall prevention during and after hospitalization (85, 86). Finally, the weak predictive performance of KAB-centered models suggests that future risk stratification and prevention efforts should integrate richer clinical and environmental data. Incorporation of validated inpatient fall risk tools, objective mobility measures (e.g., gait speed, Timed Up and Go), delirium screening, medication burden metrics, and ward-level environmental and staffing variables may yield improved prediction and more actionable intervention targets (71, 87). In the Chinese context, where rural–urban differences in health outcomes and healthcare utilization persist despite broad insurance coverage, multilevel approaches that incorporate social determinants and post-discharge environment may be particularly relevant (36, 88).

This study has several strengths, including a large sample size with dyadic patient–caregiver participation, comprehensive measurement of KAB domains alongside education quality, health literacy, and communication barriers, and the use of a cascade framework to identify specific implementation points where education fails. The progressive modeling strategy also provides transparent evidence that the examined domains, as operationalized here, have limited explanatory value for fall/near-fall events in this setting. Several limitations should temper interpretation. Most fundamentally, this study operationalized a KAB-centered conceptual model that, while appropriate for community-based fall prevention research, exhibits inherent theoretical limitations when applied to acute inpatient fall events. The consistently poor predictive performance (AUC = 0.577, pseudo-*R*^2^ = 0.013) is not merely a statistical issue but reflects that the measured distal determinants (knowledge, attitudes, behaviors, education quality, health literacy) are conceptually mismatched to the proximal, rapidly evolving clinical factors that dominate inpatient fall risk over short hospitalization periods. Effective inpatient fall prediction and prevention require integration of acute clinical variables (delirium status, medication burden, objective mobility measures, staffing adequacy, environmental hazards) with patient engagement constructs, rather than relying on education-centric frameworks alone. The cross-sectional design precludes causal inference and is susceptible to reverse causality, particularly regarding health literacy and education exposure. Outcomes were based on self-report and included near-falls, which may be variably interpreted, potentially introducing misclassification; future studies should triangulate events using incident reports and clinical documentation. Several key inpatient fall determinants were not measured, including delirium, medication classes (e.g., sedatives), objective functional status, staffing ratios, ward environmental hazards, and timing of events, all of which may explain the low model discrimination. Self-reported behaviors may be influenced by social desirability, particularly among caregivers. Finally, the single-center design may limit generalizability across China’s heterogeneous hospital systems, although the large sample and inclusion of both patients and caregivers increase the relevance of findings for similar tertiary settings.

## 5 Conclusion

This large hospital-based study revealed that while fall prevention knowledge and attitudes were comparable between older adult patients and caregivers, critical gaps persisted regarding high-risk factors such as prior fall history and environmental modifications. Despite caregivers demonstrating higher preventive behaviors, the study identified systemic implementation failures, including inconsistent teach-back verification and low adherence to provider recommendations. Crucially, while traditional knowledge and education quality metrics failed to predict outcomes, health literacy and communication comfort emerged as significant independent predictors of fall reporting, suggesting that patient engagement capacity fundamentally influences event recognition. These results challenge the sufficiency of education-centric models in acute care, indicating that inpatient falls are driven by multifactorial clinical and environmental determinants rather than knowledge deficits alone. Consequently, prevention strategies must shift from passive information delivery to system-embedded, literacy-sensitive interventions that enforce comprehension verification and integrate active patient engagement with objective clinical risk protocols.

## Data Availability

The raw data supporting the conclusions of this article will be made available by the authors, without undue reservation.

## References

[1] Montero-Odasso M van der Velde N Martin FC Petrovic M Tan MP Ryg J . World guidelines for falls prevention and management for older adults: a global initiative. Age Ageing. (2022) 51:afac205. doi: 10.1093/ageing/afac205, 36178003 PMC9523684

[2] Chen Y Dai F Huang S Qi D Peng C Zhang A . Global, regional, and national burden of falls among older adults: findings from the Global Burden of Disease Study 2021 and projections to 2040. npj Aging. (2025) 11:85. doi: 10.1038/s41514-025-00275-4, 41068164 PMC12511421

[3] Florence CS Bergen G Atherly A Burns E Stevens J Drake C. Medical costs of fatal and nonfatal falls in older adults. J Am Geriatr Soc. (2018) 66:693–8. doi: 10.1111/jgs.15304, 29512120 PMC6089380

[4] Haddad YK Miller GF Kakara R Florence C Bergen G Burns ER . Healthcare spending for non-fatal falls among older adults, USA. Inj Prev. (2024) 30:272–6. doi: 10.1136/ip-2023-045023, 39029927 PMC11445707

[5] Chaabna K Jithesh A Khawaja S Aboughanem J Mamtani R Cheema S. The epidemiology of unintentional falls among older people in the Middle East and North Africa: a systematic review and meta-analysis. J Glob Health. (2025) 15:04072. doi: 10.7189/jogh.15.04072, 40084526 PMC11907375

[6] Wang X Wang R Zhang D. Bidirectional associations between sleep quality/duration and multimorbidity in middle-aged and older people Chinese adults: a longitudinal study. BMC Public Health. (2024) 24:708. doi: 10.1186/s12889-024-17954-8, 38443848 PMC10916205

[7] Guirguis-Blake JM Perdue LA Coppola EL Bean SI. Interventions to prevent falls in older adults: updated evidence report and systematic review for the US Preventive Services Task Force. JAMA. (2024) 332:58–69. doi: 10.1001/jama.2024.4166, 38833257 PMC13552531

[8] Lee SH Yu S. Effectiveness of multifactorial interventions in preventing falls among older adults in the community: a systematic review and meta-analysis. Int J Nurs Stud. (2020) 106:103564. doi: 10.1016/j.ijnurstu.2020.103564, 32272282

[9] Rahimi S Khankeh HR Ebadi A Mohammadian B Arsalani N Fallahi-Khoshknab M . Development and validation of the Fall Risk Assessment Scale for patients in rehabilitation hospitals: a methodological study. Health Sci Rep. (2024) 7:e70040. doi: 10.1002/hsr2.70040, 39507677 PMC11538047

[10] Menard HE Castro-Pearson S Dahle N Edmonds SW Kozitza BJ Webb JJ . Fall risk assessment in acute rehabilitation: comparison of two assessment tools. Rehabil Nurs J. (2025) 50:24–32. doi: 10.1097/rnj.0000000000000487, 39774056

[11] Gillespie LD Robertson MC Gillespie WJ Sherrington C Gates S Clemson LM . Interventions for preventing falls in older people living in the community. Cochrane Database Syst Rev. (2012) 2012:CD007146. doi: 10.1002/14651858.CD007146.pub3, 22972103 PMC8095069

[12] Pillay J Gaudet LA Saba S Vandermeer B Ashiq AR Wingert A . Falls prevention interventions for community-dwelling older adults: systematic review and meta-analysis of benefits, harms, and patient values and preferences. Syst Rev. (2024) 13:289. doi: 10.1186/s13643-024-02681-3, 39593159 PMC11590344

[13] Stevens JA Lee R. The potential to reduce falls and avert costs by clinically managing fall risk. Am J Prev Med. (2018) 55:290–7. doi: 10.1016/j.amepre.2018.04.035, 30122212 PMC6103639

[14] Johnston YA Reome-Nedlik C Parker EM Bergen G Wentworth L Bauer M. Preventing falls among older adults in primary care: a mixed methods process evaluation using the RE-AIM framework. Gerontologist. (2023) 63:511–22. doi: 10.1093/geront/gnac111, 35917287 PMC10258889

[15] Hill AM Vaz S Francis-Coad J Flicker L Morris ME Weselman T. ‘You Just Struggle on Your Own’: exploring older people and their caregivers’ perspectives about falls prevention education in hospitals. Int J Older People Nursing. (2024) 19:e12628. doi: 10.1111/opn.12628, 38995867

[16] Stevens JA Sleet DA Rubenstein LZ. The influence of older adults’ beliefs and attitudes on adopting fall prevention behaviors. Am J Lifestyle Med. (2018) 12:324–30. doi: 10.1177/1559827616687263, 32063817 PMC6993092

[17] Manirajan P Sivanandy P Ingle PV. Enhancing knowledge, attitude, and perceptions towards fall prevention among older adults: a pharmacist-led intervention in a primary healthcare clinic, Gemas, Malaysia. BMC Geriatr. (2024) 24:309. doi: 10.1186/s12877-024-04930-5, 38566052 PMC10988811

[18] Alfaro Hudak KM Adibah N Cutroneo E Liotta M Sanghera A Weeks-Gariepy T . Older adults’ knowledge and perception of fall risk and prevention: a scoping review. Age Ageing. (2023) 52:afad220. doi: 10.1093/ageing/afad220, 38016017

[19] Laing SS Silver IF York S Phelan EA. Fall prevention knowledge, attitude, and practices of community stakeholders and older adults. J Aging Res. (2011) 2011:395357. doi: 10.4061/2011/395357, 21915377 PMC3170803

[20] Albasha N Curtin C McCullagh R Cornally N Timmons S. Staff perspectives on fall prevention activities in long-term care facilities for older residents: “Brief but often” staff education is key. PLoS One. (2024) 19:e0310139. doi: 10.1371/journal.pone.0310139, 39250475 PMC11383231

[21] Ong MF Soh KL Saimon R Wai MW Mortell M Soh KG. Fall prevention education to reduce fall risk among community-dwelling older persons: a systematic review. J Nurs Manag. (2021) 29:2674–88. doi: 10.1111/jonm.13434, 34331491 PMC9291009

[22] Park Y Kim SR Seo HJ Cho J. Health literacy in fall-prevention strategy: a scoping review. Asian Nurs Res. (2024) 18:532–44. doi: 10.1016/j.anr.2024.10.011, 39549947

[23] Shaw L Kiegaldie D Farlie MK. Education interventions for health professionals on falls prevention in health care settings: a 10-year scoping review. BMC Geriatr. (2020) 20:460. doi: 10.1186/s12877-020-01819-x, 33167884 PMC7653707

[24] Mahmoodi R Hassanzadeh A Rahimi M. Health literacy and its dimensions in elderly people in Farsan city, Iran. J Educ Health Promot. (2021) 10:362. doi: 10.4103/jehp.jehp_149_21, 34761048 PMC8552271

[25] Lee DC McDermott F Hoffmann T Haines TP. They will tell me if there is a problem’: limited discussion between health professionals, older adults and their caregivers on falls prevention during and after hospitalization. Health Educ Res. (2013) 28:1051–66. doi: 10.1093/her/cyt091, 24045410

[26] Chen QF Ni C Jiang Y Chen L Liao H Gao J . Global burden of disease and its risk factors for adults aged 70 and older across 204 countries and territories: a comprehensive analysis of the Global Burden of Disease Study 2021. BMC Geriatr. (2025) 25:462. doi: 10.1186/s12877-025-06095-1, 40604507 PMC12220231

[27] Sui L Lv Y Feng KX Jing FJ. Burden of falls in China, 1992–2021 and projections to 2030: a systematic analysis for the Global Burden of Disease Study 2021. Front Public Health. (2025) 13:1538406. doi: 10.3389/fpubh.2025.1538406, 40190758 PMC11968356

[28] Ye P Er Y Wang H Fang L Li B Ivers R . Burden of falls among people aged 60 years and older in mainland China, 1990–2019: findings from the Global Burden of Disease Study 2019. Lancet Public Health. (2021) 6:e907–18. doi: 10.1016/s2468-2667(21)00231-0, 34838197 PMC8646839

[29] Wang K Chen M Zhang X Zhang L Chang C Tian Y . The incidence of falls and related factors among Chinese elderly community residents in six provinces. Int J Environ Res Public Health. (2022) 19:14843. doi: 10.3390/ijerph192214843, 36429561 PMC9690932

[30] Yeong UY Tan SY Yap JF Choo WY. Prevalence of falls among community-dwelling elderly and its associated factors: a cross-sectional study in Perak, Malaysia. Malays Fam Physician. (2016) 11:7–14. 28461842 PMC5405326

[31] Long S Hu L Luo Y Li Y Ding F. Incidence and risk factors of falls in older adults after discharge: a prospective study. Int J Nurs Sci. (2023) 10:23–9. doi: 10.1016/j.ijnss.2022.12.010, 36860715 PMC9969166

[32] Wu H Ouyang P. Fall prevalence, time trend and its related risk factors among elderly people in China. Arch Gerontol Geriatr. (2017) 73:294–9. doi: 10.1016/j.archger.2017.08.009, 28910753

[33] Huang H Zhou J Zhao M Li W Schwebel DC Rao Z . Unintentional fall mortality by place, sex, and age group among older Chinese adults, 2010–2021. J Glob Health. (2024) 14:04170. doi: 10.7189/jogh.14.04170, 39325920 PMC11893140

[34] Horton K Dickinson A. The role of culture and diversity in the prevention of falls among older Chinese people. Can J Aging. (2011) 30:57–66. doi: 10.1017/s0714980810000826, 21401976

[35] Lin WQ Chen JM Yuan LX Wang JY Sun SY Sun MY . Association between abdominal obesity indices and falls among older community-dwellers in Guangzhou, China: a prospective cohort study. BMC Geriatr. (2024) 24:732. doi: 10.1186/s12877-024-05319-0, 39232713 PMC11373189

[36] Ying M Wang S Bai C Li Y. Rural-urban differences in health outcomes, healthcare use, and expenditures among older adults under universal health insurance in China. PLoS One. (2020) 15:e0240194. doi: 10.1371/journal.pone.0240194, 33044992 PMC7549821

[37] Chen X Lin Z Gao R Yang Y Li L. Prevalence and associated factors of falls among older adults between urban and rural areas of Shantou City, China. Int J Environ Res Public Health. (2021) 18:7050. doi: 10.3390/ijerph18137050, 34280986 PMC8297296

[38] Zhao D Li J Fu P Hao W Yuan Y Yu C . What role does activity engagement play in the association between cognitive frailty and falls among older adults? Evidence from rural Shandong, China. Gerontology. (2020) 66:593–602. doi: 10.1159/000510639, 33045703

[39] Heng H Jazayeri D Shaw L Kiegaldie D Hill AM Morris ME. Hospital falls prevention with patient education: a scoping review. BMC Geriatr. (2020) 20:140. doi: 10.1186/s12877-020-01515-w, 32293298 PMC7161005

[40] Hill AM Loo CY Coulter S Watson C Vaz S Morris ME . Health professional perceptions of delivering hospital falls prevention education—a qualitative study. West J Nurs Res. (2025) 47:348–55. doi: 10.1177/01939459251319078, 39945422 PMC11993816

[41] Shrestha P Fick DM. Family caregiver’s experience of caring for an older adult with delirium: a systematic review. Int J Older People Nursing. (2020) 15:e12321. doi: 10.1111/opn.12321, 32374518

[42] Sultana M Taylor C McPhee D Chandler A Hyde H Yekinni I . Reducing falls in inpatient older adults: quality improvement initiative. J Alzheimers Dis. (2025) 107:86–91. doi: 10.1177/13872877251360031, 40685630 PMC12361677

[43] Kiyoshi-Teo H Northrup-Snyder K Robert Davis M Garcia E Leatherwood A Seiko Izumi S. Qualitative descriptions of patient perceptions about fall risks, prevention strategies and self-identity: analysis of fall prevention Motivational Interviewing conversations. J Clin Nurs. (2020) 29:4281–8. doi: 10.1111/jocn.15465, 32810908

[44] Petrocchi S Filipponi C Schulz PJ. A longitudinal application of the actor partner interdependence model extended mediations to the health effects of dyadic support. PLoS One. (2021) 16:e0254716. doi: 10.1371/journal.pone.0254716, 34280225 PMC8289016

[45] Boltz M Kuzmik A Resnick B BeLue R. Recruiting and retaining dyads of hospitalized persons with dementia and family caregivers. West J Nurs Res. (2022) 44:319–27. doi: 10.1177/01939459211032282, 34382886 PMC9472232

[46] Nasreddine ZS Phillips NA Bédirian V Charbonneau S Whitehead V Collin I . The Montreal Cognitive Assessment, MoCA: a brief screening tool for mild cognitive impairment. J Am Geriatr Soc. (2005) 53:695–9. doi: 10.1111/j.1532-5415.2005.53221.x, 15817019

[47] Faul F Erdfelder E Lang AG Buchner A. G*Power 3: a flexible statistical power analysis program for the social, behavioral, and biomedical sciences. Behav Res Methods. (2007) 39:175–91. doi: 10.3758/bf03193146, 17695343

[48] Hill KD Day L Haines TP. What factors influence community-dwelling older people’s intent to undertake multifactorial fall prevention programs? Clin Interv Aging. (2014) 9:2045–53. doi: 10.2147/cia.s72679, 25473276 PMC4251660

[49] Peter D Robinson P Jordan M Lawrence S Casey K Salas-Lopez D. Reducing readmissions using teach-back: enhancing patient and family education. J Nurs Adm. (2015) 45:35–42. doi: 10.1097/nna.0000000000000155, 25479173

[50] Morris NS MacLean CD Chew LD Littenberg B. The Single Item Literacy Screener: evaluation of a brief instrument to identify limited reading ability. BMC Fam Pract. (2006) 7:21. doi: 10.1186/1471-2296-7-21, 16563164 PMC1435902

[51] Sudore RL Landefeld CS Pérez-Stable EJ Bibbins-Domingo K Williams BA Schillinger D. Unraveling the relationship between literacy, language proficiency, and patient-physician communication. Patient Educ Couns. (2009) 75:398–402. doi: 10.1016/j.pec.2009.02.019, 19442478 PMC4068007

[52] World Health Organization (WHO). WHO global report on falls prevention in older age2008. Geneva: World Health Organization (WHO) (2008).

[53] Schoene D Wu SM Mikolaizak AS Menant JC Smith ST Delbaere K . Discriminative ability and predictive validity of the timed up and go test in identifying older people who fall: systematic review and meta-analysis. J Am Geriatr Soc. (2013) 61:202–8. doi: 10.1111/jgs.12106, 23350947

[54] White IR Royston P Wood AM. Multiple imputation using chained equations: issues and guidance for practice. Stat Med. (2011) 30:377–99. doi: 10.1002/sim.4067, 21225900

[55] Hill AM Francis-Coad J Vaz S Morris ME Flicker L Weselman T . Implementing falls prevention patient education in hospitals—older people’s views on barriers and enablers. BMC Nurs. (2024) 23:633. doi: 10.1186/s12912-024-02289-x, 39256815 PMC11389421

[56] Zhang K Yang Z Zhang X Li L. Comparison of falls and risk factors among older adults in urban villages, urban and rural areas of Shantou, China. Heliyon. (2024) 10:e30536. doi: 10.1016/j.heliyon.2024.e30536, 38737229 PMC11087945

[57] Zhang L Ding Z Qiu L Li A. Falls and risk factors of falls for urban and rural community-dwelling older adults in China. BMC Geriatr. (2019) 19:379. doi: 10.1186/s12877-019-1391-9, 31888516 PMC6937656

[58] Haines TP Lee DC O’Connell B McDermott F Hoffmann T. Why do hospitalized older adults take risks that may lead to falls? Health Expect. (2015) 18:233–49. doi: 10.1111/hex.12026, 23194444 PMC5060778

[59] Adam HL Giroux CM Eady K Moreau KA. A qualitative study of patients’ and caregivers’ perspectives on educating healthcare providers. Can Med Educ J. (2021) 12:7–16. doi: 10.36834/cmej.71541, 34567301 PMC8463222

[60] Alnazly EK. The impact of an educational intervention in caregiving outcomes in Jordanian caregivers of patients receiving hemodialysis: a single group pre-and-post test. Int J Nurs Sci. (2018) 5:144–50. doi: 10.1016/j.ijnss.2018.03.007, 31406816 PMC6626206

[61] Upsher R Dommett E Carlisle S Conner S Codina G Nobili A . Improving reporting standards in quantitative educational intervention research: introducing the CLOSER and CIDER checklists. J New Approaches Educ Res. (2025) 14:2. doi: 10.1007/s44322-024-00022-9, 39916891 PMC11801185

[62] Ng IK. Adopting the information-knowledge-attitude-practice model for transforming health behaviors in medical practice. Oman Med J. (2024) 39:e264. doi: 10.5001/omj.2024.120, 39559554 PMC11571051

[63] Dashti S Abadibavil D Roozbeh N. Evaluating e-health literacy, knowledge, attitude and practice regarding COVID-19 prevention and self-protection among Iranian students: a cross-sectional online survey. BMC Med Educ. (2022) 22:148. doi: 10.1186/s12909-022-03210-3, 35248025 PMC8897549

[64] Ames AJ. Measuring response style stability across constructs with item response trees. Educ Psychol Meas. (2022) 82:281–306. doi: 10.1177/00131644211020103, 35185160 PMC8850762

[65] Boateng GO Neilands TB Frongillo EA Melgar-Quiñonez HR Young SL. Best practices for developing and validating scales for health, social, and behavioral research: a primer. Front Public Health. (2018) 6:149. doi: 10.3389/fpubh.2018.00149, 29942800 PMC6004510

[66] Cooper S Schmidt BM Sambala EZ Swartz A Colvin CJ Leon N . Factors that influence parents’ and informal caregivers’ views and practices regarding routine childhood vaccination: a qualitative evidence synthesis. Cochrane Database Syst Rev. (2021) 10:CD013265. doi: 10.1002/14651858.CD013265.pub2, 34706066 PMC8550333

[67] Latkin CA Edwards C Davey-Rothwell MA Tobin KE. The relationship between social desirability bias and self-reports of health, substance use, and social network factors among urban substance users in Baltimore, Maryland. Addict Behav. (2017) 73:133–6. doi: 10.1016/j.addbeh.2017.05.005, 28511097 PMC5519338

[68] Ebrahim N Ras J November R Leach L. Medication use and fall risk among older adults in long-term care facilities: a cross-sectional analysis. South Afr J Sports Med. (2025) 37:v37i1a20605. doi: 10.17159/2078-516X/2025/v37i1a20605, 40959593 PMC12435277

[69] Li S Wang J Ren L Ye P Niu W Yu M . Health literacy and falls among community-dwelling older people in China: is there a sex difference? Aging Clin Exp Res. (2024) 36:148. doi: 10.1007/s40520-024-02788-6, 39023697 PMC11258050

[70] Weidmann AE Proppé GB Matthíasdóttir R Tadić I Gunnarsson PS Jónsdóttir F. Medication-induced causes of delirium in patients with and without dementia: a systematic review of published neurology guidelines. Int J Clin Pharm. (2025) 47:606–23. doi: 10.1007/s11096-024-01861-4, 39969659 PMC12125033

[71] Locklear T Kontos J Brock CA Holland AB Hemsath R Deal A . Inpatient falls: epidemiology, risk assessment, and prevention measures. A narrative review. HCA Healthc J Med. (2024) 5:517–25. doi: 10.36518/2689-0216.1982, 39524960 PMC11547277

[72] Najafpour Z Godarzi Z Arab M Yaseri M. Risk factors for falls in hospital in-patients: a prospective nested case control study. Int J Health Policy Manag. (2019) 8:300–6. doi: 10.15171/ijhpm.2019.11, 31204446 PMC6571495

[73] Lakbala P Bordbar N Fakhri Y. Root cause analysis and strategies for reducing falls among inpatients in healthcare facilities: a narrative review. Health Sci Rep. (2024) 7:e2216. doi: 10.1002/hsr2.2216, 38946779 PMC11211207

[74] Flanders WD Klein M Darrow LA Strickland MJ Sarnat SE Sarnat JA . A method to detect residual confounding in spatial and other observational studies. Epidemiology. (2011) 22:823–6. doi: 10.1097/EDE.0b013e3182305dac, 21968772 PMC3233361

[75] Zheng Y Lamoureux EL Chiang PP Cheng CY Anuar AR Saw SM . Literacy is an independent risk factor for vision impairment and poor visual functioning. Invest Ophthalmol Vis Sci. (2011) 52:7634–9. doi: 10.1167/iovs.11-7725, 21873660

[76] Savitz DA Wellenius GA. Can cross-sectional studies contribute to causal inference? It depends. Am J Epidemiol. (2023) 192:514–6. doi: 10.1093/aje/kwac037 35231933

[77] Hitcho EB Krauss MJ Birge S Claiborne Dunagan W Fischer I Johnson S . Characteristics and circumstances of falls in a hospital setting: a prospective analysis. J Gen Intern Med. (2004) 19:732–9. doi: 10.1111/j.1525-1497.2004.30387.x, 15209586 PMC1492485

[78] Lindberg DS Prosperi M Bjarnadottir RI Thomas J Crane M Chen Z . Identification of important factors in an inpatient fall risk prediction model to improve the quality of care using EHR and electronic administrative data: a machine-learning approach. Int J Med Inform. (2020) 143:104272. doi: 10.1016/j.ijmedinf.2020.104272, 32980667 PMC8562928

[79] Mao A Su J Ren M Chen S Zhang H. Risk prediction models for falls in hospitalized older patients: a systematic review and meta-analysis. BMC Geriatr. (2025) 25:29. doi: 10.1186/s12877-025-05688-0, 39810076 PMC11730783

[80] Talevski J Wong Shee A Rasmussen B Kemp G Beauchamp A. Teach-back: a systematic review of implementation and impacts. PLoS One. (2020) 15:e0231350. doi: 10.1371/journal.pone.0231350, 32287296 PMC7156054

[81] Holcomb J Ferguson GM Thornton L Highfield L. Development, implementation, and evaluation of Teach Back curriculum for community health workers. Front Med. (2022) 9:918686. doi: 10.3389/fmed.2022.918686, 36405583 PMC9669070

[82] Choopani A Arabloo J Arkian SH Vatankhah S. Identification of the contextual factors influencing the successful implementation of in-service training policies for the health workforce in Iran. BMC Med Educ. (2024) 24:1365. doi: 10.1186/s12909-024-06336-8, 39593039 PMC11590634

[83] Pillay J Riva JJ Tessier LA Colquhoun H Lang E Moore AE . Fall prevention interventions for older community-dwelling adults: systematic reviews on benefits, harms, and patient values and preferences. Syst Rev. (2021) 10:18. doi: 10.1186/s13643-020-01572-7, 33422103 PMC7797084

[84] Correia T Martins MM Barroso F Pinho L Longo J Valentim O. Patient and family-centered care to promote inpatient safety: an exploration of nursing care and management processes. Nurs Rep. (2025) 15:260. doi: 10.3390/nursrep15070260, 40710954 PMC12299392

[85] Ahmed WE Fakhry SF Mohamed Badran FM. Bedside handover training and its effects on nurses’ knowledge and compliance. BMC Nurs. (2025) 24:1472. doi: 10.1186/s12912-025-04075-9, 41299617 PMC12687478

[86] Wilandika A Pandin MGR Yusuf A. The roles of nurses in supporting health literacy: a scoping review. Front Public Health. (2023) 11:1022803. doi: 10.3389/fpubh.2023.1022803, 37663836 PMC10469320

[87] Greene BR O’Donovan A Romero-Ortuno R Cogan L Scanaill CN Kenny RA. Quantitative falls risk assessment using the timed up and go test. IEEE Trans Biomed Eng. (2010) 57:2918–26. doi: 10.1109/tbme.2010.2083659, 20923729

[88] Li J Shi L Liang H Ding G Xu L. Urban-rural disparities in health care utilization among Chinese adults from 1993 to 2011. BMC Health Serv Res. (2018) 18:102. doi: 10.1186/s12913-018-2905-4, 29426313 PMC5807772

[89] Qasim M Awan UA Afzal MS Saqib MAN Siddiqui S Ahmed H. Dataset of knowledge, attitude, practices and psychological implications of healthcare workers in Pakistan during COVID-19 pandemic. Data Brief. (2020) 32:106234. doi: 10.1016/j.dib.2020.106234, 32895632 PMC7462453

[90] Awan UA Naeem W Khattak AA Mahmood T Kamran S Khan S et al. An exploratory study of knowledge, attitudes, and practices toward HPV associated anal cancer among Pakistani population. Front Oncol. (2023) 13:1257401. doi: 10.3389/fonc.2023.1257401, 37954070 PMC10637352

[91] Awan UA Guo X Khattak AA Hassan U Khan S. Economic crises and cancer care in Pakistan-timely action saves lives. Lancet. (2024) 403:613–614. doi: 10.1016/S0140-6736(23)01380-6, 38368005

[92] Awan UA Song Q Ciombor KK Toriola AT Choi J Su T . Demographic and Clinicopathologic Factors Associated With Colorectal Adenoma Recurrence. JAMA Netw Open. (2026) 9:e2556853. doi: 10.1001/jamanetworkopen.2025.56853, 41637071 PMC12873766

